# Requiem of Olympic ethics and sports’ independence: A panel-data socio-cultural analysis

**DOI:** 10.1371/journal.pone.0335957

**Published:** 2025-11-06

**Authors:** Fabio Zagonari

**Affiliations:** Dipartimento di Scienze per la Qualità della Vita, Università di Bologna, C.so d’Augusto, Rimini, Italy; Çanakkale Onsekiz Mart University: Canakkale Onsekiz Mart Universitesi, TÜRKIYE

## Abstract

This paper suggests a theoretical model (a production function) and an empirical model (Stochastic Frontier Analysis) to *empirically* evaluate the main impacts of socio-cultural contexts on the effectiveness of some sport policies and to address some main *methodological* problems of sport sociology. As for methods, I identified 2 governmental ethics to and through sport (national pride NP, social cohesion SC), by measuring achievements in terms of alternative indexes based on Olympic medals (gold, total) from 1994 to 2024. I applied panel-data, by focusing on 4 alternative estimations (individual and collective variables for both NP and SC). I introduced 2 sport policies (a quantitative policy aimed at SC, a qualitative policy aimed at NP), by distinguishing *cultural* approaches to body in terms of 5 different secular ethics (Aristotle, Husserl, Deleuze, Heidegger, Descartes) and 5 different religious ethics (Buddhism, Christianism, Hinduism, Islam, Judaism). I referred to income level and income inequality (i.e., GDP and Gini index), to depict alternative *social* contexts. I applied country dummies, to represent alternative historical and institutional contexts. As for results, if governments pursue SC, there is significant consonance with more communitarian religions and dissonance with more individualistic religions (to a greater extent at a collective level), whereas religions do not affect the effectiveness of sport policies if governments pursue NP. If governments pursue NP, there is significant consonance with secular body approaches deemphasising mind over body (at an individual level only), whereas if governments pursue SC, there is significant consonance with Deleuze, Heidegger and Descartes and dissonance with Husserl (to a smaller extent at a collective level). In summary, this paper *empirically* highlights the social and cultural contexts affecting some sport policies, by providing a quantitative *methodology* to identify groups of countries with institutional or historical peculiarities, to be studied by sport sociology with complementary qualitative methodologies.

## 1. Introduction

There are many theoretical papers on ethics *to* sport (i.e., on government approaches or athletes’ attitudes toward sports); for example, Twietmeyer et al. [1] on Christian ethics, Tak et al. [2] on Confucianism, and Frias [3] on Protestant ethics. There are few empirical papers on ethics to sport; for example, De Waegeneer et al. [4] on moral intentions. Note that research on ethics to sport has recently focused on sport integrity (i.e., sport stakeholders upholding a range of moral values such as honesty, sportsmanship, respect, and trustworthiness in fulfilling their sport organisational roles, namely, professional responsibility, as well as within wider society, namely, personal responsibility, to ensure a safe, fair, and inclusive on-field and in-competition activity) by taking an individual and psychological perspective (e.g., Robertson & Constandt [5], Quartiroli & Wagstaff [6]).

There are many theoretical papers on ethics *through* sport (i.e., social or individual ethics acquired from sports); for example, Avner et al. [7] on sports work, Pankow et al. [8] on psychological skills, Debognies et al. [9] on disadvantaged youths. There are few empirical papers on ethics through sport; for example, Uğraş et al. [10] on dedication and Quartiroli et al. [11] on self-care. Note that research on ethics through sport has recently focused on sport education (i.e., sport students learning a range of moral values such as patience, tolerance, friendship, mutual respect, and excellence to address the most common threats to sustainable development, including global economic polarisation, deepening of social in-equalities, neglect of human rights, and destruction of the global environment), by taking an international and social perspective (e.g., Park & Lim [12]; Robertson et al. [13]).

Sport sociology (or sociology of sport) studies the social impacts of sport (Wenner [14]; Giulianotti & Thiel [15]), with a knowledge domain of European and North American sociologists of sport (Tian & Wise [16]). For example, within *theoretical* and qualitative papers, Howe et al. [17] focus on race discrimination, Pavlenko [18] on transgender discrimination, Smith et al. [19] and McMillan [20] on climate change, Lehtonen et al. [21] on indigenous inclusion, Pulleiro Méndez [22] and Shen & Fan [23] on *national pride*, Moustakas [24] on *social cohesion*. Next, within *empirical* and quantitative papers, Silva et al. [25] focus on sport participation and SWB, but it relies on a survey with 511 college students in Portugal; Campillo-Sanchez et al. [26] focus on UN sustainable development goals, but it is based on the analysis of 7 papers; McSweeney & Nakamura [27] focus on cultural integration, but it relies on the analysis of 26 studies. In particular, within the articles on elite sports, Zare & Geczi [28] focus on *national pride*, but it is based on an open-ended survey with 32 respondents. Note that the methodologies applied by empirical papers are often inadequate in sport sociology (Olive et al. [29]).

Sport policy (or policy of sport) studies alternative processes (Parent & Jurbala [30]) to implement policies with alternative contents (Lindsey et al. [31]) in the sporting space, often to produce social impacts (Dowling & Harris [32]; Ouyang et al. [33]; Moradi et al. [34]). For example, within *theoretical* and qualitative papers, Piller & Nagel [35] focus on sustainable development, Pielke Jr & Harris [36] on management and governance, Posberg [37] on transgender discrimination, Sam et al. [38] on sport integrity, Garcia & Meier [39] on federations’ autonomy, Grix & Brannagan [40] on sportswashing, Viollet et al. [41] on voluntary or not-for-profit organisations, Terason & Pahasing [42] on *national pride*, Raikonnen & Hedman [43] and Moustakas [44] on *social cohesion*. Next, within *empirical* and quantitative papers, Engdal et al. [45] focus on volunteers’ engagement to achieve sport inclusion, but it relies on the analysis of 27 studies; Pankowiak et al. [46] focus on the development and implementation of effective national sport policies/systems to optimise Paralympic success, but it is based on 23 semi-structured interviews with national Paralympic sport managers from the United Kingdom, Australia, France and Canada; Volf et al. [47] focus on sport participation and physical activity, but it relies on the analysis of 22 papers; Chatzigianni & Mallen [48] on global governance and environmental sustainability, but it is based on content/thematic analysis of reports by 34 sport organisations. In particular, within the articles on elite sports, Zheng et al. [49] focus on *national pride,* but it is based on 21 semi-structured interviews about 3 sport disciplines in Taiwan. Note that the methodologies applied by empirical papers are often inadequate in sport policy (Mountifield [50]).

However, Zagonari [51] shows that winning medals is a production rather than a stochastic process (i.e., requiem of Olympic ethics and sports’ independence), with alternative elite sport policies aiming at national pride or social cohesion. Moreover, Jedlicka et al. [52] shows that alternative *social* contexts impact on effectiveness of sport policies. Finally, Zagonari [53] shows that different body approaches (i.e., having a body vs. being a body) impact differently on happiness and health, with alternative religious or secular *cultural* approaches beneficially or detrimentally affecting happiness and health at individual or collective levels.

In other words, two main gaps can be identified in the literature: there are no empirical papers on social impacts of elite sport policies in alternative individual or collective social contexts and religious or secular cultural contexts; there are no panel-data analysis based on theoretical models applied to complete samples at a country level for individual and collective socio-cultural features.

The *purpose* of this paper is to bridge these two gaps (i.e., one topical gap and one methodological gap), by evaluating the main impacts of sociocultural contexts on the effectiveness of specified sport policies (i.e., national pride and social cohesion) and by addressing the main highlighted methodological problems of sport sociology (i.e., meso or micro analyses applied to small samples and unfounded in theoretical models).

To do so, I identified 2 governmental ethics to and through sport (i.e., national pride NP, social cohesion SC), by measuring achievements in terms of alternative indexes based on Olympic medals (i.e., gold medals per capita GM, total medals per capita TM). Moreover, I applied an estimation method (i.e., a panel data Stochastic Frontier Analysis SFA), by focusing on 4 alternative estimations (i.e., individual IND and collective COL variables for both NP and SC). Finally, I introduced 2 sport policies (i.e., a quantitative policy based on the total number of athletes and disciplines aimed at social cohesion POLC, a qualitative policy based on the variations’ coefficient of athletes across disciplines aimed at national pride POLP), by distinguishing cultural approaches to body in terms of 5 different secular SEC ethics (i.e., Aristotle ARI, Husserl HUS, Deleuze DEL, Heidegger HEI, Descartes DES) and 5 different religious REL ethics (i.e., Buddhism BUD, Christianism CHR, Hinduism HIN, Islam ISL, Judaism JUD).

Note that I referred to income level and income inequality (i.e., Gross Domestic Product per capita GDP and the Gini Index INE) to depict alternative social contexts, as used in the literature (e.g., Yeh et al. [54]). Moreover, I referred to the percentages of believers in the 5 main religions (i.e., BUD, CHR, HIN, ISL, JUD), the percentages of people practicing indirect, direct, contact and team sports (i.e., ARI, HUS, DEL, HEI, respectively) and the education expenditures per student in 3 educational levels (i.e., primary EEP, secondary EES and tertiary EET) for DES to depict alternative cultural contexts at an individual level, whereas I referred to the percentages of believers above 5%, the percentages of people practicing sports above 5% and the gross enrolment rates in 3 educational levels (i.e., primary GEP, secondary GES and tertiary GET) for DES to depict alternative cultural contexts at a collective level. Finally, I relied on country dummies to depict other historical or institutional country peculiarities, as used in the literature (e.g., Ogwang & Cho [55]).

Thus, the objective of the present study is to estimate to what extent qualitative or quantitative sport policies are affected by alternative social and cultural contexts, where positive or negative statistically significant impacts on sport policies mean that consonance or dissonance prevail on average, since my sample includes *all* athletes participating in Olympic games from 1994 to 2024 (i.e., 8 winter and 8 summer editions).

In other words, the research questions can be summarised as follows: 1) do social contexts affect sport policies aimed at national pride? 2) do cultural contexts affect sport policies aimed at national pride? 3) do social contexts affect sport policies aimed at social cohesion? 4) do cultural contexts affect sport policies aimed at social cohesion?

Note that I improved the SPLISS (Sport Policy factors Leading to International Sport Success) framework (Henry et al., 2020 [56]) by combining macro-level theorising of elite sport policies (i.e., governmental policies in a macro-level model) with the identification of country specificities in terms of social and cultural features (i.e., average variables) and historical or institutional features (i.e., country dummies).

In summary, the *topical* contribution of the present paper is twofold: it finds that impacts of social contexts on SC and NP are statistically significant and not significant, respectively, at an individual level to a greater extent than at a collective level; it finds that impacts of some REL and SEC cultural contexts on both SC and NP are larger at collective than at individual levels, respectively. In addition, the *methodological* contribution of the present paper is twofold: SFA is adequate to identify casual relationships, by lagging independent variables (e.g., averages over the two previous years), whenever overall inefficiency is non-significant; panel data analysis is adequate to distinguish groups of countries, by paving the way to complementary qualitative analyses, whenever common features (e.g., historical or institutional features) are identified.

Note that NBIC technologies (i.e., nanotechnology, biotechnology, information technology, and cognitive science) are against social cohesion (e.g., Aggerholm [57]). Moreover, I will disregard studies on, and close to, the literature on sports medicine (e.g., Zurc [58] on acceptance of health risks by athletes), sports management (e.g., Ribeiro et al. [59] on Olympic Games), sports education (e.g., Bakhtiyarova et al. [60] on ideals, values, principles of Olympism) and sports regulation (e.g., Muñoz et al. [61] on governance of sports organisations). Finally, doping and testosterone are against national pride (e.g., Park & Ok [62]).

The structure of the present paper is as follows. Section 2 suggests the comprehensive theoretical framework, by introducing an empirical methodology to complete the literature. Section 3 details the empirical model. Section 4 constructs the dataset, by considering summer and winter Olympic games from 1994 to 2024. Section 5 details the estimations of the empirical model. Section 6 discusses the achievements of the empirical model combined with respect to the research questions, by highlighting strengths and weaknesses of the methodology suggested in the present paper. Section 7 discusses the achievements of SFA with respect to the research purposes, by highlighting the topical and methodological successes of the present paper.

## 2. The theoretical framework

From the literature on ethics to and through sport, one can obtain 2 goals (national pride, social cohesion) (e.g., [55]) and 2 indexes (gold medals, total medals) (e.g., [63]). I will complete this theoretical framework by introducing SFA with country dummies to represent the relationships between sport policies and socio-cultural contexts, by considering country specificities (e.g., [51]). Moreover, from the literature on ethics to and through sport, one can obtain 2 main independent social variables (GDP and INE) (e.g., [54]). I will complete this theoretical framework with 2 sport policies (POLP, POLC) and 2 groups of cultural independent variables (REL, SEC) [64]. In particular, I will define POLP (i.e., a qualitative assessment of the governmental sport policy assumed to be directed to national pride) as the variations’ coefficient of athletes across disciplines and POLC (i.e., a quantitative assessment of the governmental sport policy assumed to be directed to social cohesion) as the total number of athletes across disciplines [65]. Note that I used the variations’ coefficients across categories to standardise with respect to the number of athletes for each country, since there is a maximum given number of athletes for each discipline and the number of disciplines for each category if fixed. Finally, from the literature on body approaches (e.g., [53]), one can obtain many alternative REL and SEC ethics (Table S1 in S1 File Supplementary Materials SM provides a theoretical framework conceptually schematising the literature on the main REL and SEC approaches to body (i.e., “HAVING a body” vs. “BEING a body”) in sport disciplines and physical activities). I will distinguish REL approaches into 5 main religions (i.e., percentages of believers in BUD, CHR, HIN, ISL, JUD at a national level), whereas I will distinguish SEC approaches into 5 main philosophies (i.e., ARI, HUS, DEL, HEI as percentages of people practicing indirect, direct, contact and team sports at a national level, respectively) and DES as the education expenditures per capita in 3 education levels in individual estimations and DES as gross enrolment rates in 3 education levels in collective estimations.

Table 1 summarises the governmental goals, output indices and input variables in a theoretical framework, by stressing expected positive or negative impacts of different input variables on alternative output indices.

**Table 1:** The theoretical framework and the expected impacts. Abbreviations: GM = gold medals, TM = total medals, + = expected positive impact, - = expected negative impact. Notes: REL will be split into BUD, CHR, HIN, ISL, JUD in individual (all percentages) and collective (percentages above 5) estimations; SEC will be split into ARI, HUS, DEL, HEI in individual (all percentages) and collective (percentages above 5) estimations and into EEP, EES, EET in individual estimations and GEP, GES GET in collective estimations for DES; POL will be used as POLP and POLC.

Governmental goals	(Output) Indexes	(Input) Variables					Estimations	Results
		POL	GDP	INE	REL	SEC		
National pride	Lexicographic GM	–	+/-	+/-	+/-	+/-	at an individual level	Table 3
							at a collective level	Table 5
Social cohesion	Sum TM	+	+/-	+/-	+/-	+/-	at an individual level	Table 4
							at a collective level	Table 6

**Note** that some impacts are expected to be positive. For example, a larger income level could favour larger funds to sports [66], although I will identify which governmental goal is more affected by the per capita income level. Similarly, a larger quantitative or qualitative education is likely to make sport funds more productive [67], although I will specify which education level is more productive. Moreover, some impacts are expected to be negative. For example, a negative REL represents the absence of confrontative ethics in most religions [68], although I will specify which religions are less confrontative. Finally, some impacts could be either positive or negative as dependent on the governmental goal under consideration. For example, a positive impact of income inequality could depict larger sport achievements in more inequal countries, due to the larger social redemption attached to sport [69], but a negative impact of INE could depict larger sport achievements in more equal countries, due to the larger opportunities for gifted people [70]. Similarly, I will use total population to standardize medals (i.e., to compare small and large countries) rather than as an independent variable, since finding an Olympic athlete is a matter of quality (i.e., gifted people) rather than of quantity (i.e., young people) [71,72]. Consequently, a positive impact of POLC could depict the effectiveness of a governmental sport policy based on a large number of disciplines, but a negative POLP could depict the effectiveness of a governmental sport policy focused on a small number of disciplines. All these expected impacts will be verified in Section 5.

Some *methodological* observations are worthy here. First, I did not use a dummy variable for the hosting country and a variable for the time trend, although Zagonari [51] provides all estimations with these additional variables. I did not refer to democracy, population aged 20–34 years, freedom, perceived corruption, since they are not significant in the literature. I did not use weighted medals, since relative weights (e.g., 3, 2, 1 for gold, silver and bronze medals, respectively) are arbitrary. I did not refer to climate conditions, since they are depicted by country dummies. Second, in governmental sport policies, I did not distinguish countries where governments finance sports directly (e.g., governmental federations), countries where governments finance sports indirectly (e.g., athletes employed in specific armies), and countries where governments do not finance sports (e.g., athletes enrolled in private colleges). However, governmental goals to sport are similar. In governmental sport policies, I did not distinguish professional from non-professional sports. However, individual ethics to sport are similar. Third, countries without medals are excluded [73]. Sport achievements are assumed to be pursued, by referring to a stochastic production function based on specified inputs. A single goal is depicted as independent variable [74]. A complete ranking of countries in terms of efficiency is provided, by estimating significance and value of *all* country dummies.

Note that there is a limit to the number of participants so large countries are underestimated. Moreover, POLC is a sufficient but not necessary condition for a sport diffusion at a national level, although it changes over time and it estimates also quality of sport federations (i.e., number of athletes in each category) as well as quantity of sports (i.e., category with at least one athlete). Finally, Gini Index measures an outcome of both REL and SEC ethics at a country level (i.e., many cultural and institutional contexts).

## 3. The empirical model

SFA applied to Olympic medals refers to the well-grounded production theory: Y = f(X), where Y is the achieved production level and X is the vector of used production factors. In particular, SFA can depict the Olympic medals (M_i,t_) within random-effects panel-data models as follows:


$${LnM}_{i,t}=\alpha_{POL}{LnPOL}_{i,t}+\beta_{GDP}{GDP}_{i,t}+\beta_{INE}{INE}_{i,t}+\beta_{REL}{REL}_{i,t}+\beta_{SEC}{SEC}_{i,t}{+D}_{i}+\zeta_{i,t}+\xi_{i,t}$$

With:


$$\zeta_{i,t}∽N(\mu ,\sigma \text{u})\text{and}\xi_{i,t}∽N(\text{0},\sigma \text{v})$$

Where Ln is the natural logarithm and D_i_ are dummy variables catching the country specificities. Note that coefficients of production factors represent increasing returns to scale or decreasing returns to scale production functions if they are larger than 1 or smaller than 1, respectively; ζ_i,t_ depicts the level of efficiency of observation i at time t (truncated at 0 with mean μ and variance σu); and ξ_i,t_ represents the idiosyncratic error (independently and identically distributed with mean 0 and variance σv) [75]. In other words, it is assumed a Cobb-Douglas production function M = f(POL) which can be shifted up or down by income level, income inequality, religious and secular ethics, once the country specificities other than these cultural features are caught by the country dummy variables (i.e., all constant terms are assumed to be depicted by variables other than POL).

A *methodological* observation is worthy here. I will use POLP for NP by referring to GM and I will use POLC for SC by referring to TM [51], although GM is an indirect measure of NP (i.e., other factors affect the expected link between GM and NP) and TM is an indirect measure of SC (i.e., other factors affect the expected link between TM and SC).

## 4. The dataset

Section 1 suggested the adoption of a representative individual perspective at a country level, Section 2 suggested 2 governmental goals (i.e., NP and SC), 2 output indices (i.e., GM and TM) and 4 input indices (i.e., GDP, INE, REL, SEC), while Section 3 developed an empirical model based on a production process as a theoretical model. In this section, I will describe the per capita variables used to estimate the 2 empirical models in alternative social and cultural contexts (The dataset is available at https://osf.io/ek3zf). In particular, the number of Olympic medals and the number of Olympic athletes for each discipline are obtained from the Olympic dataset (www.olympic.org). Moreover, the percentages of believers in all religions are obtained from World Religions dataset (www.worldreligions.org). Finally, GDP per capita (in current USD), INE (the Gini Index), qualitative education (i.e., EEP, EES, EET as the education expenditures per student in current USD in primary, secondary and tertiary education) and quantitative education (i.e., GEP, GES, GET as the gross enrolment rates in percentages in primary, secondary and tertiary education) are obtained from the World Bank dataset.

Note that I focused on income inequality, since fairness is the crucial ethical concept in sport, whereas I disregarded gender discrimination, since ethics to and through sport is the same for distinct male and female sporting disciplines.

Table 2 summarises the main statistics of the used variables, by considering summer and winter Olympic games from 1996 to 2024 (8 winter and 8 summer editions). Note that winter and summer Olympic games were in the same year in 1992 and before 1992 [76].

**Table 2:** Statistics of the used variables. Notes: all variables are per country and per Olympic event. Abbreviations: IND = ARI, DIR = HUS, CON = DEL, TEA = HEI, N = Number of, PC = per capita, PS = per student.

Name	Meaning	Unit	Mean	SD	MAX	MIN
GM	Gold Medals	N	10.90	10.75	163	0
TM	Total Medals	N	23.15	14.38	323	0
IND	Indirect Olympic sports	%	0.22	0.92	57.83	0.00
DIR	Direct Olympic sports	%	0.40	2.69	183.62	0.00
CON	Contact Olympic sports	%	0.06	0.16	9.97	0.00
TEA	Team Olympic sports	%	0.19	0.69	49.16	0.00
POLP	A qualitative policy aimed at national pride	Variation’s coefficient of athletes across disciplines	1.37	0.60	2	0
POLC	A quantitative policy aimed at social cohesion	Total number of athletes across disciplines	61.55	66.34	856	1
GDP	Gross Domestic Product PC	Current Thousand USD	15.897	24.140	208.835	0.337
INE	Inequality	Gini Index in [0, 1]	0.38	0.16	0.65	0.24
BUD	Believers in Buddhism	%	0.12	0.14	0.87	0.00
CHR	Believers in Christianism	%	0.57	0.39	0.99	0.00
HIN	Believers in Hinduism	%	0.07	0.09	0.74	0.00
ISL	Believers in Islam	%	0.31	0.34	1.00	0.00
JUD	Believers in Judaism	%	0.03	0.06	0.74	0.00
EEP	Expenditure in Primary Education PS	Current Thousand USD	3.416	2.303	23.203	0.014
EES	Expenditure in Secondary Education PS	Current Thousand USD	4.046	2.675	22.872	0.034
EET	Expenditure in Tertiary Education PS	Current Thousand USD	7.293	5.793	105.095	0.001
GEP	Primary Gross Enrolment rate	%	1.01	0.38	1.52	0.00
GES	Secondary Gross Enrolment rate	%	0.82	0.41	1.63	0.00
GET	Tertiary Gross Enrolment rate	%	0.39	0.30	1.50	0.00
POP	Population	Million	41	153	1423	0

Some *methodological* observations are worthy here. First, the number of medals and athletes for team sports are larger than for individual sports. Second, I used the average of the two previous values for each variable other than medals (i.e., for each independent variable), since Olympic games take place in the first months and in the middle months of the Olympic year for winter and summer editions, respectively. Indeed, the obviously unbalanced sample I used (i.e., few countries win at least one medal of the same type in all Olympic events) did not allow me to empirically implement stationary and causality tests. Third, I used the proportion of athletes across disciplines for each country in 2020 also for 2024, since data for 2024 show medals and total athletes only.

## 5. The empirical results

Section 3 suggested an empirical model (i.e., SFA) based on a theoretical model (i.e., a production function at a country level), while Section 4 described the per capita variables to estimate the empirical model in alternative social and cultural contexts. In this section, I will estimate this empirical model by referring to the alternative goals and indices summarised in Table 1. In particular, Table 3 and Table 4 present results for POLP aimed at NP and POLC aimed at SC in individual estimations, respectively, whereas Table 5 and Table 6 present results for POLP aimed at NP and POLC aimed at SC in collective estimations, respectively.

**Table 3:** SFA applied to GM and POLP with an INDIVIDUAL perspective. No. observations = 601, No. groups = 100, non-significant μ (average inefficiency) = −0.656, non-significant η (increasing inefficiency) = −0.492, σu2 (similarity between countries) = 0.250, σv2 (similarity between countries over time) = 0.635. Bold = significant at 95%. CONS = estimated constant.

LnGM	Coefficient	Std. err.	z	P > z	[95% conf.	interval]
LnPOLP	−.9658205	.1797975	−5.37	**0.000**	−1.318217	−.6134239
GDP	−4.47e-06	6.84e-06	−0.65	0.513	−.0000179	8.93e-06
INE	.0769567	1.076951	0.07	0.943	−2.033829	2.187742
BUD	8.430892	19.17684	0.44	0.660	−29.15502	46.0168
CHR	−.7158876	.6304781	−1.14	0.256	−1.951602	.5198268
HIN	30.89937	25.889	1.19	0.233	−19.84214	81.64089
ISL	−1.118021	4.640968	−0.24	0.810	−10.21415	7.978108
JUD	29.72777	34.10115	0.87	0.383	−37.10925	96.5648
ARI	2.193711	.9414937	2.33	**0.020**	.348417	4.039004
HUS	−.4267613	.339408	−1.26	0.209	−1.091989	.2384663
DEL	10.38504	3.636456	2.86	**0.004**	3.257717	17.51236
HEI	.8113917	.7820165	1.04	0.299	−.7213325	2.344116
EEP	−.0162196	.0636026	−0.26	0.799	−.1408784	.1084391
EES	.0192496	.0573263	0.34	0.737	−.093108	.1316071
EET	.0090339	.0269493	0.34	0.737	−.0437857	.0618535
CONS	−1.20432	.9209055	−1.31	0.191	−3.009262	.6006215

**Table 4:** SFA applied to TM and POLC with an INDIVIDUAL perspective. No. observations = 853, No. groups = 128, non-significant μ (average inefficiency) = −1.159, non-significant η (increasing inefficiency) = −0.996, σu2 (similarity between countries) = 0.234, σv2 (similarity between countries over time) = 0.379. Bold = significant at 95%. CONS = estimated constant.

LnTM	Coefficient	Std. err.	z	P > z	[95% conf.	interval]
LnPOLC	1.072912	.0487392	22.01	**0.000**	.9773854	1.16844
GDP	−9.87e-06	4.67e-06	−2.11	**0.035**	−.000019	−7.09e-07
INE	−2.124001	.7185703	−2.96	**0.003**	−3.532373	−.7156289
BUD	−10.91952	8.80011	−1.24	0.215	−28.16742	6.328382
CHR	−.1285075	.4394092	−0.29	0.770	−.9897337	.7327186
HIN	.3932409	3.032383	0.13	0.897	−5.55012	6.336602
ISL	1.107346	.6610274	1.68	**0.094**	−.188244	2.402936
JUD	−14.24161	17.59043	−0.81	0.418	−48.71822	20.235
ARI	.7455012	.518752	1.44	0.151	−.2712341	1.762236
HUS	−.6060674	.185519	−3.27	**0.001**	−.969678	−.2424569
DEL	4.653175	2.038545	2.28	**0.022**	.6577011	8.648649
HEI	1.354285	.4104958	3.30	**0.001**	.5497285	2.158842
EEP	−.0535809	.0399867	−1.34	0.180	−.1319533	.0247915
EES	.0538042	.0360434	1.49	0.135	−.0168396	.1244479
EET	.0250267	.0095889	2.61	**0.009**	.0062328	.0438206
CONS	−3.086898	.6684895	−4.62	0.000	−4.397113	−1.776682

**Table 5:** SFA applied to GM and POLP with a COLLECTIVE perspective. No. observations = 601, No. groups = 100, non-significant μ (average inefficiency) = −0.365, non-significant η (increasing inefficiency) = −0.532, σu2 (similarity between countries) = 0.201, σv2 (similarity between countries over time) = 0.635. Bold = significant at 95%. CONS = estimated constant.

LnGM	Coefficient	Std. err.	z	P > z	[95% conf.	interval]
LnPOLP	−1.178129	.1681322	−7.01	**0.000**	−1.507663	−.8485963
GDP	3.01e-07	5.44e-06	0.06	0.956	−.0000104	.000011
INE	.1694792	1.081357	0.16	0.875	−1.949941	2.2889
BUDcol	5.514241	26.83927	0.21	0.837	−47.08976	58.11824
CHRcol	.2871811	1.841304	0.16	0.876	−3.321708	3.89607
HINcol	43.52711	111.7927	0.39	0.697	−175.5825	262.6368
ISLcol	1.617944	2.77828	0.58	0.560	−3.827384	7.063272
JUDcol	41.65735	41.45038	1.00	0.315	−39.58389	122.8986
ARIcol	.8977055	.7907983	1.14	0.256	−.6522307	2.447642
HUScol	−.0636127	.3146447	−0.20	0.840	−.6803049	.5530795
DELcol	2.429391	3.55473	0.68	0.494	−4.537753	9.396534
HEIcol	.0086731	.7239033	0.01	0.990	−1.410151	1.427497
GEP	−1.392002	.6498427	−2.14	**0.032**	−2.66567	−.1183335
GES	−.0710987	.4011284	−0.18	0.859	−.8572959	.7150986
GET	−.4668596	.295254	−1.58	0.114	−1.045547	.1118277
CONS	−.3635843	1.807718	−0.20	0.841	−3.906647	3.179478

**Table 6:** SFA applied to TM and POLC with a COLLECTIVE perspective. No. observations = 853, No. groups = 128, non-significant μ (average inefficiency) = −0.784, non-significant η (increasing inefficiency) = −1.039, σu2 (similarity between countries) = 0.209, σv2 (similarity between countries over time) = 0.381. Bold = significant at 95%. CONS = estimated constant.

LnTM	Coefficient	Std. err.	z	P > z	[95% conf.	interval]
LnPOLC	1.121747	.0433735	25.86	**0.000**	1.036736	1.206757
GDP	−4.45e-06	3.46e-06	−1.29	0.199	−.0000112	2.34e-06
INE	−2.052042	.7213446	−2.84	**0.004**	−3.465851	−.6382321
BUDcol	−24.12195	14.37035	−1.68	**0.093**	−52.28732	4.043422
CHRcol	1.773287	1.181206	1.50	0.133	−.5418338	4.088407
HINcol	−52.83422	40.2264	−1.31	0.189	−131.6765	26.00808
ISLcol	3.155997	1.74859	1.80	**0.071**	−.2711756	6.583171
JUDcol	−13.34115	20.05518	−0.67	0.506	−52.64857	25.96627
ARIcol	.5721587	.4495818	1.27	0.203	−.3090055	1.453323
HUScol	−.4139561	.1779756	−2.33	**0.020**	−.7627818	−.0651304
DELcol	2.962267	1.974194	1.50	0.133	−.9070816	6.831616
HEIcol	.8849273	.395129	2.24	**0.025**	.1104887	1.659366
GEP	−.2555276	.2774904	−0.92	0.357	−.7993989	.2883436
GES	−.1818726	.2577111	−0.71	0.480	−.6869771	.3232319
GET	.1831045	.1972872	0.93	0.353	−.2035713	.5697803
CONS	−4.434657	1.134111	−3.91	0.000	−6.657474	−2.21184

Note that I used only POLP as a sport policy for NP and only POLC as a sport policy for SC, as suggested by Zagonari [51]. Moreover, I measured ARI, HUS, DEL, HEI in terms of DIR, IND, CON, TEA, as suggested by Zagonari [53]. In particular, it is assumed that individuals practice a sport discipline or a physical activity if they espouse their philosophical (SEC) or theological (REL) approaches to body; the number of Olympic athletes in a country for each sport represents its popularity in that country; and individuals practice a physical activity if it is suggested by their religions (REL) or individuals practice a sport discipline if they expect beneficial impacts on happiness, health or both (SEC). Finally, I used EEP, EES, EET as a cultural feature in individual estimations and GEP, GES, GET as a cultural feature in collective estimations, as suggested by Zagonari [77].

Table 3 shows *many* expected results. Indeed, POLP is statistically significant, whereas social contexts (i.e., GDP, INE), REL and qualitative education (i.e., EEP, EES, EET) are not statistically significant. However, it also highlights that SEC approaches deemphasising mind over body (i.e., ARI and DEL) favour the achievements of GM and NP. Note that POLP shows constant returns to scale (i.e., coefficient value close to −1).

Table 4 shows *some* expected results. Indeed, POLC is statistically significant and social contexts are statistically significant (i.e., the achieved SC is larger in poorer and more inequal countries). However, it also highlights that some REL are statistically significant (i.e., the achieved SC is larger in Muslim countries, by supporting the body’s role in social practices such as the physical postures in common prayer and the common fasting during Ramadan), some qualitative education are statistically significant (i.e., better tertiary educated people favour the achievement of SC, by supporting the prevalence of mind over body in DES in characterising human nature), and some SEC approaches to body favour (i.e., creativity of body in DEL, humanism of body in HEI) and disfavour (i.e., the experimental aspects of body in HUS) the achievements of TM and SC. Note that POLC shows constant returns to scale (i.e., coefficient value close to 1).

Note that the negatively significant impact of HUS (i.e., the most confrontational approach to body) on SC could find a theoretical background in a phenomenological interpretation of the social theory suggested by Rosa [78] (i.e., a good life as one that is rich of experiences and developed capacities). Indeed, sport as a tool for political ends (i.e., requiem of Olympic ethics with a meaningful and responsive relationship between agent and activity) could represent a lack of resonance (e.g., Hoffken, [79]), while a reduced SC (i.e., aseptic and meaningless interpersonal relations in a competitive and accelerated society) could depict an alienation from others (e.g., Mateu et al. [80]).

Comparing Table 5 with Table 3 shows that less educated people are an obstacle to the achievement of NP and GM (i.e., a negative GEP becomes statistically significant at a collective level) and body approaches are individual issues. Indeed, all SEC ethics become not statistically significant at a collective level. Thus, achieving NP is a matter of individual approaches to body (i.e., both REL and SEC cultural contexts, with irrelevant social contexts), provided that some minimum level of quantitative education is achieved, with POLP more effective at a collective level (i.e., returns to scale become increasing).

Comparing Table 6 with Table 4 shows that collective institutional factors might compensate for low individual incomes (i.e., GDP becomes not significant), a larger proportion of believers in more individualistic religions is detrimental to the achievement of SC (i.e., a negative BUD becomes statistically significant at a collective level), a larger proportion of believers in more communitarian religions is more beneficial to the achievement of SC (i.e., a statistically significant ISL becomes more positive at a collective level), and secular body approaches are individual issues. Indeed, DEL becomes not significant, the estimated coefficient for HUS becomes smaller (i.e., from −0.606 to −0.413), the estimated coefficient for HEI becomes smaller (i.e., from 1.354 to 0.884), and GET become not significant. Thus, achieving SC is a matter of REL more than SEC and COL more than IND approaches to body (i.e., cultural contexts), where social contexts characterised by income inequality favour SC, with POLC more effective at a collective level (i.e., returns to scale become increasing).

Note that SEC body approaches in Table 3 and Table 4 play complementary roles (i.e., NP vs. SC at an individual level: + ARI, + DEL vs. -HUS, + DEL, + HEI, + DES). Moreover, all estimations show that inefficiency is not statistically significant and it is not changing over time. Finally, interactions between cultural REL contexts and social contexts are highlighted in Table 4 and Table 6 (i.e., individual vs. collective level for SC: larger REL and smaller SEC impacts of cultural contexts and smaller impacts of social contexts).

In summary, the previous results provided an answer to all research questions *on average*:

1) do social contexts affect sport policies aimed at national pride? NO
2) do cultural contexts affect sport policies aimed at national pride? YES, some SEC body approaches at an individual level (i.e., + ARI, + DEL) and a quantitative education at a collective level (i.e., -GEP)
3) do social contexts affect sport policies aimed at social cohesion? YES, both income level and income inequality, to a greater extent at an individual level
4) do cultural contexts affect sport policies aimed at social cohesion? YES, some REL body approaches at a collective level to a greater extent that at individual level (i.e., -BUD, + ISL), a qualitative education at an individual level (i.e., + EET), some SEC body approaches at an individual level (i.e., -HUS, + DEL, + DES) to a greater extent than at a collective level (i.e., + HUS, + DES).

**Fig 1:**
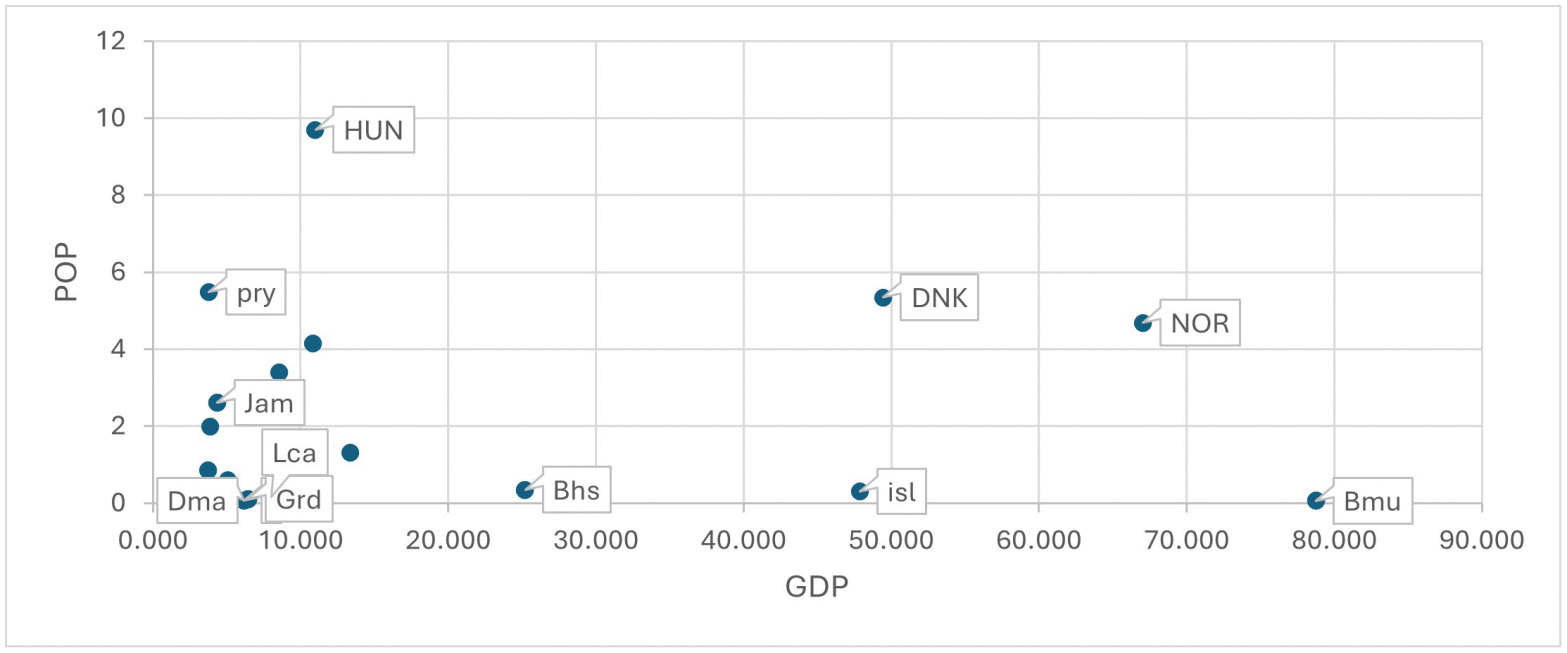
Positively above the average in TM (SC) (small codes), in GM (NP) (capital codes) and in both goals (first letter capital codes) with an INDIVIDUAL perspective.

Fig 1 identifies in terms of per capita income GDP and population POP all countries performing significantly *better* than the average (i.e., a positively significant dummy estimation) in achieving NP (capital codes), SC (small codes) or in achieving both NP and SC (first capital letter codes) in *individual* estimations. Tables S2 and S3 in S1 File Supplementary Materials SM provide all dummy estimations. Note that they are relatively small countries (i.e., POP below 10 million), where only some Caribbean countries perform better than the average in both goals.

**Fig 2:**
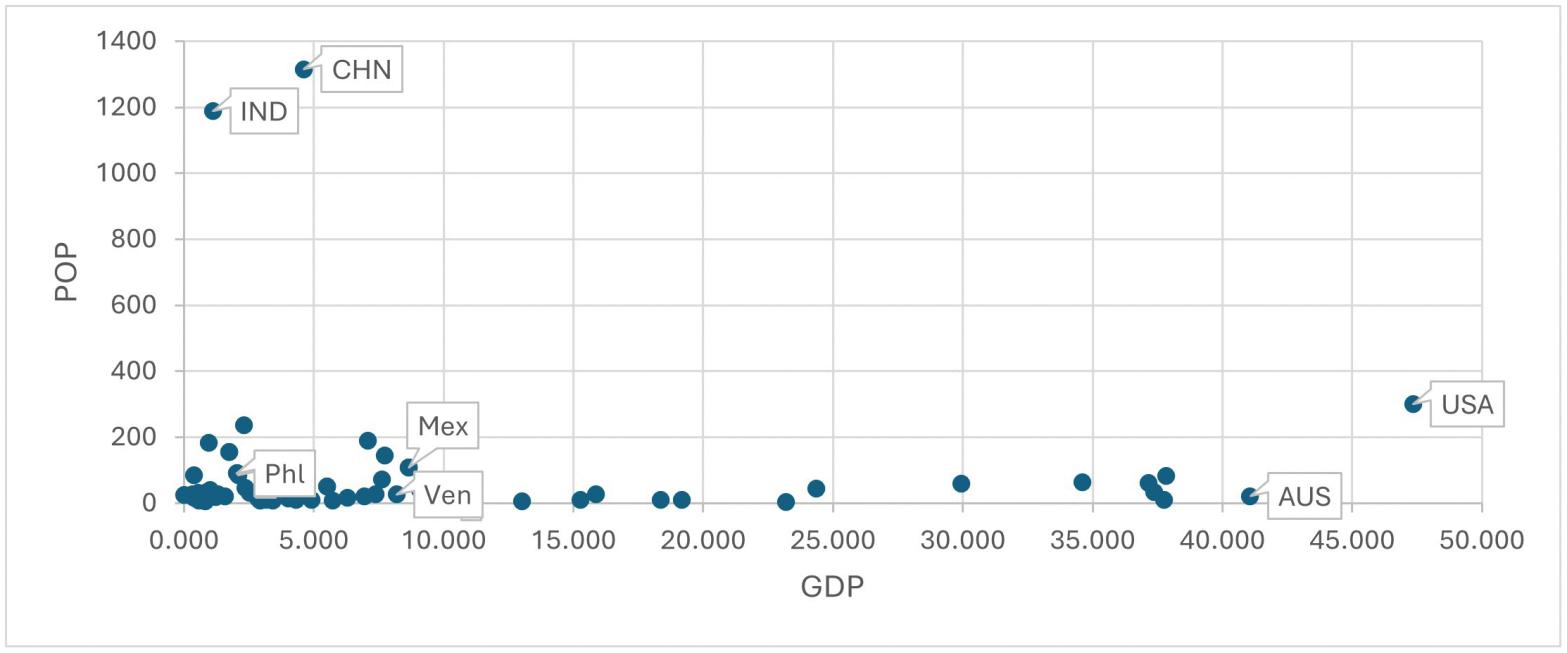
Negatively above the average in GM (NP) (capital codes) and in both goals (first letter capital codes) with an INDIVIDUAL perspective. Note: there are no small codes, since only countries negatively in both goals are negatively in TM (Social Cohesion).

Fig 2 identifies in terms of GDP and POP all countries performing significantly *worse* than the average (i.e., a negatively significant dummy estimation) in achieving NP (capital codes), SC (small codes) or in achieving both NP and SC (first capital letter codes) in *individual* estimations. Table S2 and S3 in S1 File Supplementary Materials SM provide all dummy estimations. Note that they are both large and small countries, where only some former Spanish colonies perform worse than the average in both goals.

**Fig 3:**
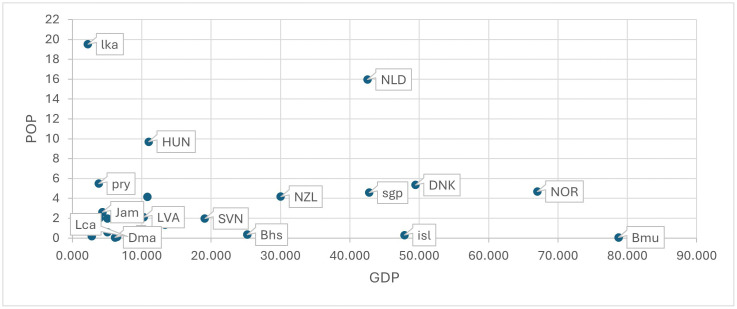
Positively above the average in TM (SC) (small codes), in GM (NP) (capital codes) and in both goals (first letter capital codes) with a COLLECTIVE perspective.

Fig 3 identifies in terms of GDP and POP all countries performing significantly *better* than the average (i.e., a positive significant dummy estimation) in achieving NP (capital codes), SC (small codes) or in achieving both NP and SC (first capital letter codes) in *collective* estimations. Comparing Fig 3 with Fig 1 shows that some countries with established social welfare systems are added (i.e., NLD, NZL, SVN, LVA), whereas Grd disappear from the Caribbean countries. Table S4 and S5 in S1 File Supplementary Materials SM provide all dummy estimations.

**Fig 4:**
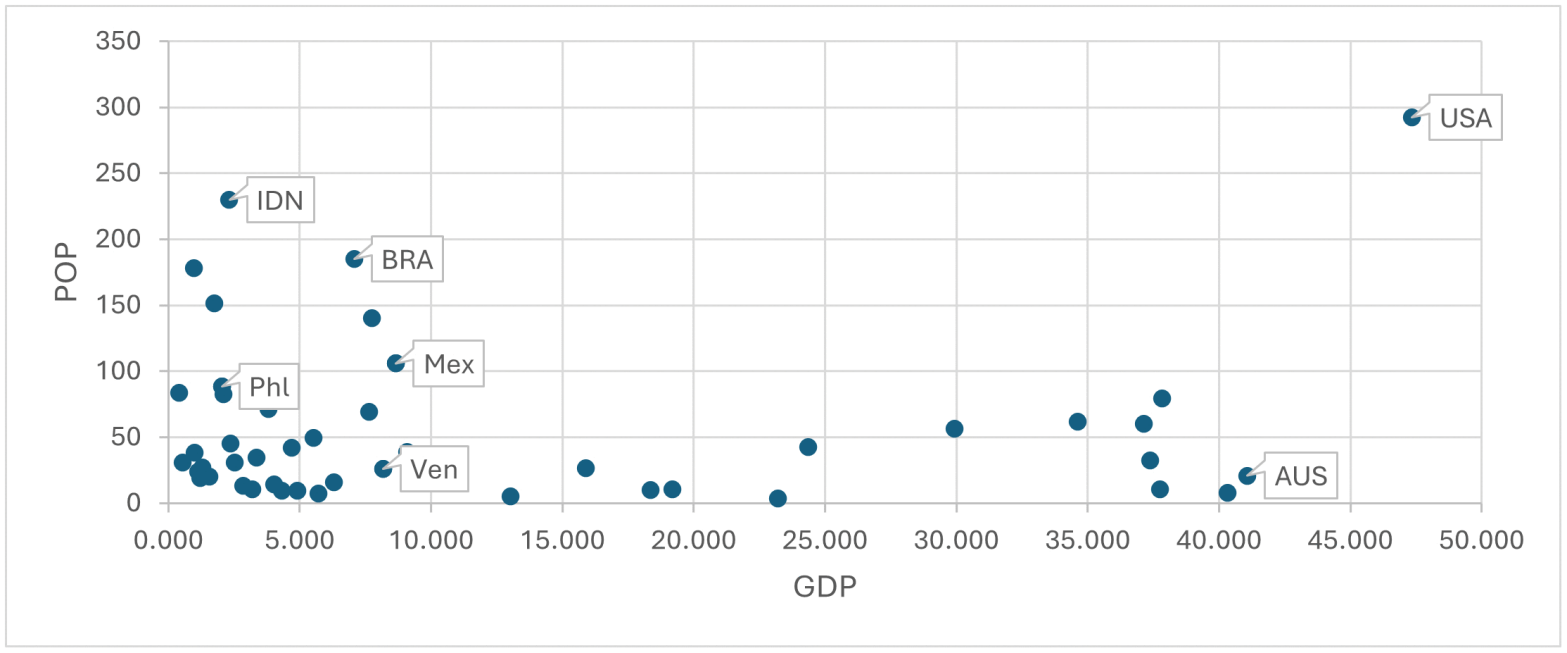
Negatively above the average in GM (NP) (capital codes) and in both goals (first letter capital codes) with a COLLECTIVE perspective. Note: there are no small codes, since only countries negatively in both goals are negatively in TM (Social Cohesion).

Fig 4 identifies in terms of GDP and POP all countries performing significantly *worse* than the average (i.e., a positive significant dummy estimation) in achieving NP (capital codes), SC (small codes) or in achieving both NP and SC (first capital letter codes) in *collective* estimations. Comparing Fig 4 with Fig 2 shows that some countries with established social welfare systems are excluded (e.g., CHN, IND, SWE, CHE), whereas all former Spanish colonies are confirmed. Table S4 and S5 in S1 File Supplementary Materials SM provide all dummy estimations.

In summary, if governments pursue social cohesion SC (i.e., by implementing POLC in Table 4 and Table 6), there is significant consonance with more communitarian religions (i.e., ISL, where the ritual purity of physical health is emphasised) and dissonance with more individualistic religions (i.e., BUD, where the rejection of both dualisms body/mind and me/world are pursued), to a greater extent if believers in these religions represent a larger proportion of the total population (i.e., at a collective level) (i.e., impacts are larger in Table 6 than in Table 4). In contrast, religions REL do not affect the effectiveness of sport policy, if governments pursue national pride NP (i.e., by implementing POLP in Table 3 and Table 5). Moreover, if governments pursue national pride NP (i.e., by implementing POLP in Table 3 and Table 5), there is significant consonance with secular body approaches deemphasising mind over body (i.e., ARI with body = mind and dualism body/mind and DEL with body > mind and no dualism body/mind is supported), at an individual level only. In contrast, if governments pursue social cohesion SC (i.e., by implementing POLC in Table 4 and Table 6), there is significant consonance with DEL (i.e., body > mind without dualism body/mind), HEI (i.e., mind > body and humanism) and DES (i.e., mind > body and individualism) and dissonance with HUS (i.e., body > mind with living and lived body), to a smaller extent if people adopting these body approaches must represent a larger proportion of the total population (i.e., at a collective level) (i.e., only HUS negatively and HEI positively impact significantly on SC in both Table 6 and in Table 4). Finally, sport policies perform better than the average in small *and* rich countries, although some institutional factors are crucial. In contrast, sport policies perform worse than the average in large *or* rich countries, although some historical factors are crucial.

## 6. Discussion

In the present paper I applied SFA to answer the 4 research questions specified in Section 1. 1) SFA applied to GM showed that social contexts do *not* affect sport policies aimed at NP, neither at an individual level nor at a collective level (Table 3 and Table 5). 2) SFA applied to GM showed that some SEC cultural contexts *positively* affect sport policies aimed at NP, although only SEC body approaches at an individual level: in particular, body approaches deemphasising body (i.e., ARI and DEL) (Table 3 and Table 5). 3) SFA applied to TM showed that social contexts (i.e., GDP and INE) negatively affect sport policies aimed at SC, to a greater extent at an individual than at a collective level (Table 4 and Table 6). 4) SFA applied to TM showed that some cultural contexts negatively affect (i.e., the SEC body approach in HUS, the REL body approach in BUD) and some cultural contexts positively affect (i.e., the SEC body approaches in DEL, HEI, DES, the REL body approach in ISL) sport policies aimed at SC, to a greater extent at an individual than at a collective level for REL and to a greater extent at a collective than at an individual level for REL (Table 4 and Table 6).

In other words, the applied methodology enabled to answer all research questions.

Note that both sport policies are pursued before the Olympic events take place (i.e., if governments pursue POLC, they are expected to make many athletes in many disciplines to achieve an Olympic level to be accepted; if governments pursue POLP, they are expected to divert sport expenditures on few disciplines) and all variables depicting social contexts (i.e., GDP, INE) and cultural contexts (i.e., REL, SEC) refer to the average values of the two years preceding the Olympic years. Consequently, the present paper estimated causal relationships (i.e., a significant coefficient estimated for an independent variable POL, GDP, INE, REL, SEC highlights its *causal* impact on the dependent variable under consideration NP, SC). In other words, I applied a practical method to the *full* longitudinal sample of Olympic countries, by emulating the main logic behind the Granger causality concept (i.e., the cause happens prior to its effect, the cause has unique information about the future values of its effect) to address the possible causality problem. Moreover, returns to scale are larger in collective than in individual estimations, for both NP and SC. Finally, the relationships between GM and NP as well between TM and SC are theoretically supported (e.g., Baim et al. [81], Mutz & Gerke [82]), but not empirically measured yet (i.e., a measurement problem of latent variables might be relevant for NP and SC). However, I used standardised dependent variables (i.e., Olympic medals per country are constrained above by the total number of medals and the maximum number of athletes per country in each sport discipline), I estimated differences of averages and I used a full sample, while the many (unbiased) plausible results validate the suggested theoretical model as an innovative methodology to interpret these interdisciplinary issues (Bulbulia [83]). Additionally, future analyses could be performed, by developing structural models (e.g., VanderWeele & Vansteelandt [84]) with Olympic medals and other factors potentially affecting NP and SC (e.g., right-wing partisanship, battlefield performance, xenophobic behaviours on NP; public trust, political and institutional quality, gender equity on SC) or by performing statistical invariance tests (e.g., Fisher et al. [85]) with Olympic medals and other variables measuring NP (e.g., World Values Survey, International Social Survey Programme) and SC (e.g., Indexes of Social Development, Social Capital Index) at a country level.

The main *strengths* of the present paper are as follows:

1. I applied SFA based on a production function as a theoretical model (i.e., NO black boxes).
2. I applied a panel-data analysis to countries characterised by different socio-cultural contexts, by referring to Olympic games as a case study of global ethics in many sports (i.e., NO reductionism).
3. I applied SFA as a causal model (i.e., YES causality).
4. I distinguished alternative governmental goals as well as alternative REL and SEC approaches to body (i.e., YES cultural contexts).

The main *weaknesses* of the present paper are as follows:

1. I did not use a dummy variable for hosting countries to reduce possible biases in medals in favour of hosting countries (i.e., athletes in hosting countries can have a greater access to Olympic fields, courts, rivers, …). However, SFA estimations provided in Zagonari [51] show that it is not always easier for hosting countries to obtain national pride, by winning gold medals.
2. I did not use a time trend to avoid possible biases in favour of more recent sport achievements (i.e., the number of Olympic disciplines has increased over time). However, SFA estimations provided in Zagonari [51] show that it is always harder for original Olympic countries to win the same number of total medals over time, due to the increasing number of Olympic countries.

Note that SFA does not require a balanced dataset. Consequently, adequate measures of additional features (e.g., gender inequality) could be used by SFA to show to what extent governmental goals outside sport could affect sport achievements (e.g., women over men Olympic medals). Similarly, adequate measures of alternative contexts (e.g., collectivist vs. individualist cultures) could be used by SFA to show to what extent other socio-cultural contexts could affect sport achievements (e.g., total Olympic medals).

## 7. Conclusion

The *purpose* of this paper was to empirically evaluate the impacts of sociocultural contexts on the effectiveness of specified sport policies (i.e., national pride and social cohesion) and to address the highlighted methodological problems of sport sociology (i.e., meso or micro analyses applied to small samples and unfounded in theoretical models).

The previous sections showed that I succeeded *topically*. In particular, I detailed *many* expected results (e.g., positive impacts of ARI and DEL on NP, negative impacts of GDP and HUS on SC, positive impacts of DEL and DES on SC). Moreover, I obtained *some* original results (i.e., a positive impact of ISL on SC, a negative impact of GEP on NP). Finally, I obtained *few* unexpected results (i.e., no impacts of GDP on NP). In other words, the goal of the present paper was *not* to suggest policy strategies to win Olympic medals, but to highlight the social and cultural contexts affecting the achievements of sport policies.

Actually, I did much more *methodologically*. In particular, I applied SFA at a country level (i.e., macro-analysis better than meso-analysis in including social interrelationships). Moreover, I applied panel data analysis (i.e., both results on average and specificities per country). Finally, I applied SFA to all countries (i.e., complete samples better than small samples). In other words, the goal of the present paper was to provide a quantitative methodology to identify groups of countries with institutional or historical peculiarities, to be studied by sport sociology with complementary qualitative methodologies.

Future developments could refer to doping practices in sports as the dependent variable (i.e., a negative governmental goal to be avoided), to ethics *of* or ethics *for* sports (i.e., alternative ethical perspective), or to educational protocols for sports as an independent variable (i.e., a positive governmental policy to be pursued).

## Supporting information

S1 File Supplementary materials SM. (DOCX)
