# Supplementary material for: Requiem of Olympic ethics and sports’ independence: A panel-data socio-cultural analysis
Source: PLoS One. 2025 Nov 6;20(11):e0335957. doi: 10.1371/journal.pone.0335957 (PMC12591425; doi:10.1371/journal.pone.0335957)
Supplement: S1 File — (DOCX) [file pone.0335957.s001.docx]

Supplementary Materials SM

**Table S1. Having a body vs. being a body. Abbreviations: B = Body, M = Mind, S = soul, SEC = secular, REL = religious, Dualism D1 = body/mind, Dualism D2 = me/world; DIR, IND, CON and TEA = proportions of people practicing direct, indirect, contact and team Olympic sports; BUD, CHR, HIN, ISL, JUD = percentages of believers in these religions, Comm/Inde = communitarian/independent perspectives, WO = without.**

|  | **Definitions** | **Sources** | **Issues** | | | **Remarks** | **Variables** |
| --- | --- | --- | --- | --- | --- | --- | --- |
|  |  |  | Self-identity | Individual  equilibria | Long-run  equilibria |  |  |
| I am a B |  |  |  |  |  |  |  |
| Flourishing  B = M  SEC | A set of potentials  (West) | Aristotle  (Eudaimonia)  [D1, D2] | I am my M & my S |  | B ages | My B dies | IND |
| Perspective  B < M  SEC | My view to experiences (West) | Husserl, Merleau-Ponty  [D1, D2] |  |  |  | My B dies | DIR |
| Interacting  B > M  SEC | A flow of sensations (East) | Deleuze  (B WO Organs)  [No D1 - D2] |  |  |  | My B dies | CON |
| Understanding  M > B | Pre-reflective experience (West) | Heidegger  [D1, D2] |  |  |  | My B dies | TEA |
| I have a B |  |  |  |  |  |  |  |
| From God  S > M  REL | I mortify it  (West) | CHR, ISL, JUD  [D1, D2] | I am my S | Comm | Suffering justified in God | Resurrection | Believers |
| REL | I accept it  (East) | HIN  [no D1, D2]  Yoga | I am my S | Comm | Deserved and learn from suffering | Reincarnation | Believers |
| REL | I accept it  (East) | BUD  [no D1, no D2]  Yoga | No unchanging self | Inde | Suffering from desire and ignorance | Dissolution | Believers |
| From nature  B < M  SEC | I control it  (West) | Descartes  [D1, D2] | I am my M | M is worse perceived |  | Values, ideas, … survive to B | III Education |

**Table S2. SFA applied to GM and POLP with an INDIVIDUAL perspective. No. observations = 601, No. groups = 100, non-significant μ (average inefficiency) = -0.656, non-significant η (increasing inefficiency) = −0.492, σu2 (similarity between countries) = 0.250, σv2 (similarity between countries over time) = 0.635. Bold = significant at 95%.**

| coucod | lgmpc | Coefficient | Std. err. | Z | P>z | [95% conf. | interval] |
| --- | --- | --- | --- | --- | --- | --- | --- |
|  | lpolp | -.9658205 | .1797975 | -5.37 | 0.000 | -1.318217 | -.6134239 |
|  | gdps | -4.47e-06 | 6.84e-06 | -0.65 | 0.513 | -.0000179 | 8.93e-06 |
|  | ine | .0769567 | 1.076951 | 0.07 | 0.943 | -2.033829 | 2.187742 |
|  | buds | 8.430892 | 19.17684 | 0.44 | 0.660 | -29.15502 | 46.0168 |
|  | chrs | -.7158876 | .6304781 | -1.14 | 0.256 | -1.951602 | .5198268 |
|  | hins | 30.89937 | 25.889 | 1.19 | 0.233 | -19.84214 | 81.64089 |
|  | isls | -1.118021 | 4.640968 | -0.24 | 0.810 | -10.21415 | 7.978108 |
|  | juds | 29.72777 | 34.10115 | 0.87 | 0.383 | -37.10925 | 96.5648 |
|  | eeps | -.0162196 | .0636026 | -0.26 | 0.799 | -.1408784 | .1084391 |
|  | eess | .0192496 | .0573263 | 0.34 | 0.737 | -.093108 | .1316071 |
|  | eets | .0090339 | .0269493 | 0.34 | 0.737 | -.0437857 | .0618535 |
|  | inds | 2.193711 | .9414937 | 2.33 | 0.020 | .348417 | 4.039004 |
|  | dirs | -.4267613 | .339408 | -1.26 | 0.209 | -1.091989 | .2384663 |
|  | cons | 10.38504 | 3.636456 | 2.86 | 0.004 | 3.257717 | 17.51236 |
|  | teas | .8113917 | .7820165 | 1.04 | 0.299 | -.7213325 | 2.344116 |
| DZA | di3 | -1.32722 | 4.482392 | -0.30 | 0.767 | -10.11255 | 7.458107 |
| ARG | di9 | -.0677193 | .7025428 | -0.10 | 0.923 | -1.444678 | 1.309239 |
| ARM | di10 | -.5241208 | .8963641 | -0.58 | 0.559 | -2.280962 | 1.232721 |
| AUS | di12 | .1307595 | .8482335 | 0.15 | 0.877 | -1.531748 | 1.793267 |
| AUT | di13 | .8454769 | .7184612 | 1.18 | 0.239 | -.5626811 | 2.253635 |
| AZE | di14 | -.3486158 | 4.338387 | -0.08 | 0.936 | -8.851699 | 8.154467 |
| BHS | di15 | 4.539856 | 1.15804 | 3.92 | 0.000 | 2.270139 | 6.809573 |
| BHR | di16 | .6092369 | 3.9529 | 0.15 | 0.878 | -7.138304 | 8.356778 |
| BLR | di19 | -.256404 | .7061818 | -0.36 | 0.717 | -1.640495 | 1.127687 |
| BEL | di20 | .2715133 | .7358145 | 0.37 | 0.712 | -1.170656 | 1.713683 |
| BMU | di23 | 4.692345 | 1.42941 | 3.28 | 0.001 | 1.890753 | 7.493938 |
| BWA | di27 | .6676294 | 1.415202 | 0.47 | 0.637 | -2.106115 | 3.441374 |
| BRA | di28 | -1.324296 | .7043753 | -1.88 | 0.060 | -2.704847 | .0562537 |
| BGR | di31 | -.0190932 | .8581412 | -0.02 | 0.982 | -1.701019 | 1.662833 |
| BDI | di33 | .9197338 | 1.012553 | 0.91 | 0.364 | -1.064833 | 2.904301 |
| CMR | di36 | -.0451165 | 1.163121 | -0.04 | 0.969 | -2.324792 | 2.234559 |
| CAN | di37 | -.2144732 | .8778086 | -0.24 | 0.807 | -1.934946 | 1.506 |
| CHL | di44 | -.3483709 | .8305056 | -0.42 | 0.675 | -1.976132 | 1.27939 |
| CHN | di45 | -4.690717 | 2.992014 | -1.57 | 0.117 | -10.55496 | 1.173521 |
| COL | di46 | -1.671987 | .7748604 | -2.16 | 0.031 | -3.190685 | -.1532882 |
| CRI | di50 | .8284503 | 1.017835 | 0.81 | 0.416 | -1.166469 | 2.82337 |
| CIV | di51 | -.963541 | 2.045855 | -0.47 | 0.638 | -4.973342 | 3.04626 |
| HRV | di52 | 1.59451 | .6725038 | 2.37 | 0.018 | .2764264 | 2.912593 |
| CUB | di53 | .2358395 | .7383709 | 0.32 | 0.749 | -1.211341 | 1.68302 |
| CZE | di56 | -.0965222 | .734565 | -0.13 | 0.895 | -1.536243 | 1.343199 |
| DNK | di57 | 2.18428 | .7901 | 2.76 | 0.006 | .6357121 | 3.732847 |
| DMA | di59 | 5.083649 | 1.278187 | 3.98 | 0.000 | 2.578449 | 7.588849 |
| DOM | di60 | -.936566 | .7564675 | -1.24 | 0.216 | -2.419215 | .5460832 |
| ECU | di65 | -.3522876 | .7619668 | -0.46 | 0.644 | -1.845715 | 1.14114 |
| EGY | di66 | -2.836816 | 4.09593 | -0.69 | 0.489 | -10.86469 | 5.19106 |
| EST | di70 | 1.802888 | .7668992 | 2.35 | 0.019 | .2997931 | 3.305983 |
| ETH | di72 | -1.214241 | 1.637594 | -0.74 | 0.458 | -4.423866 | 1.995385 |
| FJI | di79 | 3.405623 | 1.224516 | 2.78 | 0.005 | 1.005616 | 5.80563 |
| FIN | di80 | .8062942 | .7275218 | 1.11 | 0.268 | -.6196223 | 2.232211 |
| FRA | di82 | -.7358921 | .8481296 | -0.87 | 0.386 | -2.398196 | .9264114 |
| GEO | di86 | .0259458 | .8923508 | 0.03 | 0.977 | -1.72303 | 1.774921 |
| DEU | di87 | .3381804 | .7065667 | 0.48 | 0.632 | -1.046665 | 1.723026 |
| GIB | di89 | -177.8952 | 69.54225 | -2.56 | 0.011 | -314.1955 | -41.59487 |
| GRC | di90 | .2471287 | .7272607 | 0.34 | 0.734 | -1.178276 | 1.672534 |
| GRD | di92 | 2.930273 | 1.460159 | 2.01 | 0.045 | .0684152 | 5.792132 |
| GTM | di94 | -.4840679 | 1.015662 | -0.48 | 0.634 | -2.474729 | 1.506593 |
| HKG | di102 | -1.219843 | 3.108635 | -0.39 | 0.695 | -7.312656 | 4.87297 |
| HUN | di103 | 1.45386 | .6871688 | 2.12 | 0.034 | .1070343 | 2.800687 |
| IND | di110 | -28.35904 | 18.83976 | -1.51 | 0.132 | -65.28429 | 8.566204 |
| IDN | di111 | -3.279763 | 3.675267 | -0.89 | 0.372 | -10.48315 | 3.923627 |
| IRN | di112 | -1.430743 | 4.531367 | -0.32 | 0.752 | -10.31206 | 7.450574 |
| IRL | di114 | .7243818 | .836511 | 0.87 | 0.387 | -.9151497 | 2.363913 |
| ISR | di116 | -22.80497 | 24.83472 | -0.92 | 0.358 | -71.48013 | 25.87019 |
| ITA | di117 | -.4684438 | .6634414 | -0.71 | 0.480 | -1.768765 | .8318775 |
| JAM | di118 | 2.707684 | .7307728 | 3.71 | 0.000 | 1.275396 | 4.139972 |
| JPN | di119 | -6.604258 | 10.80288 | -0.61 | 0.541 | -27.77751 | 14.56899 |
| JOR | di120 | .3099312 | 4.420713 | 0.07 | 0.944 | -8.354508 | 8.97437 |
| KAZ | di121 | -.8738732 | 2.877189 | -0.30 | 0.761 | -6.513061 | 4.765315 |
| KEN | di122 | -.7653257 | .7267136 | -1.05 | 0.292 | -2.189658 | .6590068 |
| PRK | di124 | -1.896529 | 1.046377 | -1.81 | 0.070 | -3.94739 | .1543313 |
| KOR | di125 | -2.534262 | 4.853228 | -0.52 | 0.602 | -12.04641 | 6.97789 |
| XKX | di126 | 1.866112 | 4.265313 | 0.44 | 0.662 | -6.493748 | 10.22597 |
| LVA | di134 | 1.159852 | .7230856 | 1.60 | 0.109 | -.2573694 | 2.577074 |
| LTU | di141 | .8308132 | .7692094 | 1.08 | 0.280 | -.6768096 | 2.338436 |
| MEX | di156 | -1.785072 | .7781529 | -2.29 | 0.022 | -3.310223 | -.25992 |
| MNG | di164 | -4.663497 | 10.23489 | -0.46 | 0.649 | -24.72352 | 15.39652 |
| MAR | di166 | -1.34541 | 4.578256 | -0.29 | 0.769 | -10.31863 | 7.627806 |
| MOZ | di167 | -.7912238 | 1.126465 | -0.70 | 0.482 | -2.999056 | 1.416608 |
| NLD | di172 | .8523833 | .7605116 | 1.12 | 0.262 | -.6381921 | 2.342959 |
| NZL | di174 | .5218434 | .8953655 | 0.58 | 0.560 | -1.233041 | 2.276728 |
| NGA | di177 | -1.744085 | 1.133461 | -1.54 | 0.124 | -3.965627 | .4774576 |
| NOR | di181 | 2.392982 | .8123704 | 2.95 | 0.003 | .8007657 | 3.985199 |
| PAK | di187 | -3.085564 | 4.460851 | -0.69 | 0.489 | -11.82867 | 5.657544 |
| PAN | di189 | 1.404778 | 1.035698 | 1.36 | 0.175 | -.6251535 | 3.434709 |
| PHL | di193 | -2.732413 | .7947946 | -3.44 | 0.001 | -4.290182 | -1.174645 |
| POL | di194 | -.368512 | .6730973 | -0.55 | 0.584 | -1.687758 | .9507344 |
| PRT | di195 | -.5685829 | .773136 | -0.74 | 0.462 | -2.083902 | .9467358 |
| PRI | di198 | -.4534344 | 1.103259 | -0.41 | 0.681 | -2.615782 | 1.708913 |
| QAT | di199 | .7999932 | 3.602797 | 0.22 | 0.824 | -6.26136 | 7.861346 |
| ROU | di200 | -.1307037 | .6812935 | -0.19 | 0.848 | -1.466014 | 1.204607 |
| RUS | di201 | -.1204176 | .779968 | -0.15 | 0.877 | -1.649127 | 1.408292 |
| SRB | di208 | 1.219749 | .8029821 | 1.52 | 0.129 | -.354067 | 2.793565 |
| SGP | di211 | -2.175916 | 3.102383 | -0.70 | 0.483 | -8.256475 | 3.904643 |
| SVK | di213 | .5243532 | .6797343 | 0.77 | 0.440 | -.8079015 | 1.856608 |
| SVN | di214 | 1.275231 | .7467098 | 1.71 | 0.088 | -.1882931 | 2.738756 |
| ZAF | di218 | -1.974054 | .890137 | -2.22 | 0.027 | -3.71869 | -.2294173 |
| ESP | di222 | -.5190698 | .6897585 | -0.75 | 0.452 | -1.870972 | .832832 |
| LCA | di225 | 3.887871 | 1.129503 | 3.44 | 0.001 | 1.674085 | 6.101656 |
| SWE | di233 | .7107621 | .7444887 | 0.95 | 0.340 | -.7484089 | 2.169933 |
| CHE | di234 | .6560604 | .7650432 | 0.86 | 0.391 | -.8433967 | 2.155517 |
| SYR | di235 | -.3466676 | 4.15309 | -0.08 | 0.933 | -8.486575 | 7.79324 |
| TJK | di236 | .4485235 | 4.522126 | 0.10 | 0.921 | -8.41468 | 9.311727 |
| THA | di238 | -9.754784 | 16.71687 | -0.58 | 0.560 | -42.51924 | 23.00967 |
| TTO | di242 | -4.471734 | 6.29374 | -0.71 | 0.477 | -16.80724 | 7.86377 |
| TUN | di243 | -.6439493 | 4.515801 | -0.14 | 0.887 | -9.494757 | 8.206858 |
| TUR | di244 | -1.726151 | 4.481987 | -0.39 | 0.700 | -10.51068 | 7.058383 |
| UGA | di248 | -1.34277 | .9358549 | -1.43 | 0.151 | -3.177011 | .4914723 |
| UKR | di249 | -.9173183 | .6720364 | -1.36 | 0.172 | -2.234485 | .3998488 |
| ARE | di250 | -1.272958 | 4.00092 | -0.32 | 0.750 | -9.114617 | 6.568701 |
| GBR | di251 | -.8114468 | .7432404 | -1.09 | 0.275 | -2.268171 | .6452775 |
| USA | di252 | -.9942772 | 1.037735 | -0.96 | 0.338 | -3.028201 | 1.039647 |
| UZB | di255 | -.5391198 | 4.27405 | -0.13 | 0.900 | -8.916104 | 7.837865 |
| VEN | di257 | -1.764416 | .8403732 | -2.10 | 0.036 | -3.411518 | -.1173151 |
| VNM | di258 | -7.194603 | 9.436542 | -0.76 | 0.446 | -25.68988 | 11.30068 |
|  | _cons | -1.20432 | .9209055 | -1.31 | 0.191 | -3.009262 | .6006215 |

**Table S3. SFA applied to TM and POLC with an INDIVIDUAL perspective. No. observations = 853, No. groups = 128, non-significant μ (average inefficiency) = -1.159, non-significant η (increasing inefficiency) = −0.996, σu2 (similarity between countries) = 0.234, σv2 (similarity between countries over time) = 0.379. Bold = significant at 95%.**

| coucod | ltmpc | Coefficient | Std. err. | z | P>z | [95% conf. | interval] |
| --- | --- | --- | --- | --- | --- | --- | --- |
|  | lpolc | 1.072912 | .0487392 | 22.01 | 0.000 | .9773854 | 1.16844 |
|  | gdps | -9.87e-06 | 4.67e-06 | -2.11 | 0.035 | -.000019 | -7.09e-07 |
|  | ine | -2.124001 | .7185703 | -2.96 | 0.003 | -3.532373 | -.7156289 |
|  | buds | -10.91952 | 8.80011 | -1.24 | 0.215 | -28.16742 | 6.328382 |
|  | chrs | -.1285075 | .4394092 | -0.29 | 0.770 | -.9897337 | .7327186 |
|  | hins | .3932409 | 3.032383 | 0.13 | 0.897 | -5.55012 | 6.336602 |
|  | isls | 1.107346 | .6610274 | 1.68 | 0.094 | -.188244 | 2.402936 |
|  | juds | -14.24161 | 17.59043 | -0.81 | 0.418 | -48.71822 | 20.235 |
|  | eeps | -.0535809 | .0399867 | -1.34 | 0.180 | -.1319533 | .0247915 |
|  | eess | .0538042 | .0360434 | 1.49 | 0.135 | -.0168396 | .1244479 |
|  | eets | .0250267 | .0095889 | 2.61 | 0.009 | .0062328 | .0438206 |
|  | inds | .7455012 | .518752 | 1.44 | 0.151 | -.2712341 | 1.762236 |
|  | dirs | -.6060674 | .185519 | -3.27 | 0.001 | -.969678 | -.2424569 |
|  | cons | 4.653175 | 2.038545 | 2.28 | 0.022 | .6577011 | 8.648649 |
|  | teas | 1.354285 | .4104958 | 3.30 | 0.001 | .5497285 | 2.158842 |
| AFG | di1 | -2.1624 | 1.009973 | -2.14 | 0.032 | -4.141911 | -.1828892 |
| DZA | di3 | -4.615528 | .9482527 | -4.87 | 0.000 | -6.474069 | -2.756987 |
| ARG | di9 | -1.983442 | .5137245 | -3.86 | 0.000 | -2.990324 | -.9765607 |
| ARM | di10 | -.0708006 | .5372166 | -0.13 | 0.895 | -1.123726 | .9821247 |
| AUS | di12 | -1.488829 | .5437483 | -2.74 | 0.006 | -2.554556 | -.4231019 |
| AUT | di13 | -1.232163 | .5118454 | -2.41 | 0.016 | -2.235361 | -.2289642 |
| AZE | di14 | -2.166704 | .9098515 | -2.38 | 0.017 | -3.94998 | -.3834278 |
| BHS | di15 | 3.182427 | .7724216 | 4.12 | 0.000 | 1.668509 | 4.696346 |
| BHR | di16 | -.4010283 | .8658582 | -0.46 | 0.643 | -2.098079 | 1.296023 |
| BRB | di18 | 1.487679 | 1.017937 | 1.46 | 0.144 | -.5074408 | 3.4828 |
| BLR | di19 | -1.873256 | .4994166 | -3.75 | 0.000 | -2.852095 | -.8944175 |
| BEL | di20 | -1.536508 | .5085894 | -3.02 | 0.003 | -2.533325 | -.539691 |
| BMU | di23 | 6.145658 | 1.036363 | 5.93 | 0.000 | 4.114424 | 8.176893 |
| BWA | di27 | .8015212 | .6628263 | 1.21 | 0.227 | -.4975945 | 2.100637 |
| BRA | di28 | -3.216621 | .5264059 | -6.11 | 0.000 | -4.248357 | -2.184884 |
| BGR | di31 | -1.210907 | .4931778 | -2.46 | 0.014 | -2.177518 | -.2442961 |
| BFA | di32 | -1.784202 | .9072672 | -1.97 | 0.049 | -3.562413 | -.005991 |
| BDI | di33 | -.4531796 | .6292302 | -0.72 | 0.471 | -1.686448 | .7800889 |
| CMR | di36 | -2.037096 | .5696353 | -3.58 | 0.000 | -3.153561 | -.9206317 |
| CAN | di37 | -1.591104 | .5648143 | -2.82 | 0.005 | -2.69812 | -.4840882 |
| CHL | di44 | -1.166138 | .5470254 | -2.13 | 0.033 | -2.238288 | -.0939882 |
| CHN | di45 | -3.80396 | 1.439579 | -2.64 | 0.008 | -6.625484 | -.9824366 |
| COL | di46 | -2.998371 | .5194378 | -5.77 | 0.000 | -4.01645 | -1.980291 |
| CRI | di50 | .6544513 | .6295156 | 1.04 | 0.299 | -.5793767 | 1.888279 |
| CIV | di51 | -2.747796 | .7247303 | -3.79 | 0.000 | -4.168241 | -1.327351 |
| HRV | di52 | -.2698984 | .4949844 | -0.55 | 0.586 | -1.24005 | .7002532 |
| CUB | di53 | -.9178392 | .5311147 | -1.73 | 0.084 | -1.958805 | .1231265 |
| CYP | di55 | .7723618 | .6772578 | 1.14 | 0.254 | -.5550392 | 2.099763 |
| CZE | di56 | -1.781987 | .5318392 | -3.35 | 0.001 | -2.824372 | -.7396009 |
| DNK | di57 | -.1670843 | .5416075 | -0.31 | 0.758 | -1.228615 | .8944469 |
| DMA | di59 | 5.124074 | .9240578 | 5.55 | 0.000 | 3.312954 | 6.935194 |
| DOM | di60 | -1.344059 | .5213799 | -2.58 | 0.010 | -2.365945 | -.3221733 |
| ECU | di65 | -1.611798 | .5495057 | -2.93 | 0.003 | -2.688809 | -.5347866 |
| EGY | di66 | -5.923497 | .8845042 | -6.70 | 0.000 | -7.657093 | -4.189901 |
| ERI | di69 | .5858404 | .9053985 | 0.65 | 0.518 | -1.188708 | 2.360389 |
| EST | di70 | .4645633 | .5296425 | 0.88 | 0.380 | -.5735169 | 1.502644 |
| ETH | di72 | -2.96067 | .5616408 | -5.27 | 0.000 | -4.061465 | -1.859874 |
| FJI | di79 | .4697448 | .7712253 | 0.61 | 0.542 | -1.041829 | 1.981319 |
| FIN | di80 | -.5183577 | .5127881 | -1.01 | 0.312 | -1.523404 | .4866885 |
| FRA | di82 | -2.716334 | .5576845 | -4.87 | 0.000 | -3.809376 | -1.623293 |
| GAB | di84 | -.87048 | .7945836 | -1.10 | 0.273 | -2.427835 | .6868753 |
| GEO | di86 | -.1762586 | .5363014 | -0.33 | 0.742 | -1.22739 | .8748729 |
| DEU | di87 | -2.731373 | .5166832 | -5.29 | 0.000 | -3.744053 | -1.718692 |
| GHA | di88 | -2.460317 | .7816809 | -3.15 | 0.002 | -3.992383 | -.9282506 |
| GIB | di89 | -37.91167 | 38.85537 | -0.98 | 0.329 | -114.0668 | 38.24345 |
| GRC | di90 | -1.729543 | .5176383 | -3.34 | 0.001 | -2.744095 | -.7149903 |
| GRD | di92 | 3.673325 | .7841787 | 4.68 | 0.000 | 2.136363 | 5.210287 |
| GTM | di94 | -1.226486 | .6307673 | -1.94 | 0.052 | -2.462767 | .0097954 |
| HKG | di102 | .1559664 | 1.486768 | 0.10 | 0.916 | -2.758046 | 3.069979 |
| HUN | di103 | -.9070443 | .5130971 | -1.77 | 0.077 | -1.912696 | .0986076 |
| ISL | di105 | 2.902346 | .677446 | 4.28 | 0.000 | 1.574576 | 4.230116 |
| IND | di110 | -7.424098 | 2.39831 | -3.10 | 0.002 | -12.1247 | -2.723496 |
| IDN | di111 | -4.568083 | .8123477 | -5.62 | 0.000 | -6.160255 | -2.975911 |
| IRN | di112 | -3.854406 | .9282849 | -4.15 | 0.000 | -5.673811 | -2.035001 |
| IRL | di114 | -1.178606 | .5588616 | -2.11 | 0.035 | -2.273955 | -.0832576 |
| ISR | di116 | 8.692617 | 12.89279 | 0.67 | 0.500 | -16.57679 | 33.96203 |
| ITA | di117 | -2.748981 | .5039873 | -5.45 | 0.000 | -3.736778 | -1.761184 |
| JAM | di118 | 1.700586 | .5091864 | 3.34 | 0.001 | .7025985 | 2.698573 |
| JPN | di119 | 2.373336 | 4.975899 | 0.48 | 0.633 | -7.379246 | 12.12592 |
| JOR | di120 | -1.760311 | .9841448 | -1.79 | 0.074 | -3.689199 | .1685777 |
| KAZ | di121 | -3.492764 | .7056638 | -4.95 | 0.000 | -4.87584 | -2.109689 |
| KEN | di122 | -1.804778 | .5004832 | -3.61 | 0.000 | -2.785707 | -.8238488 |
| PRK | di124 | -2.461996 | .697472 | -3.53 | 0.000 | -3.829016 | -1.094976 |
| KOR | di125 | .5302178 | 2.259403 | 0.23 | 0.814 | -3.898131 | 4.958567 |
| XKX | di126 | -.1693602 | .9467802 | -0.18 | 0.858 | -2.025015 | 1.686295 |
| KWT | di127 | -2.471614 | .9948276 | -2.48 | 0.013 | -4.42144 | -.521788 |
| KGZ | di128 | -1.507103 | .8668167 | -1.74 | 0.082 | -3.206032 | .1918267 |
| LVA | di134 | -.1853432 | .4994982 | -0.37 | 0.711 | -1.164342 | .7936553 |
| LIE | di140 | 3.70074 | 2.2756 | 1.63 | 0.104 | -.7593538 | 8.160835 |
| LTU | di141 | -.1959738 | .5082503 | -0.39 | 0.700 | -1.192126 | .8001785 |
| MYS | di149 | -2.625565 | .8819317 | -2.98 | 0.003 | -4.354119 | -.8970106 |
| MUS | di155 | .6715488 | 1.642354 | 0.41 | 0.683 | -2.547406 | 3.890504 |
| MEX | di156 | -3.842244 | .513222 | -7.49 | 0.000 | -4.848141 | -2.836348 |
| MDA | di162 | -.3956154 | .543617 | -0.73 | 0.467 | -1.461085 | .6698543 |
| MNG | di164 | 5.130147 | 4.750543 | 1.08 | 0.280 | -4.180745 | 14.44104 |
| MNE | di165 | 1.772448 | .8716304 | 2.03 | 0.042 | .0640836 | 3.480812 |
| MAR | di166 | -4.515235 | .9380438 | -4.81 | 0.000 | -6.353767 | -2.676703 |
| MOZ | di167 | -.369352 | .8508349 | -0.43 | 0.664 | -2.036958 | 1.298254 |
| NAM | di169 | 1.554699 | .6442915 | 2.41 | 0.016 | .2919108 | 2.817487 |
| NLD | di172 | -.9888513 | .5214096 | -1.90 | 0.058 | -2.010795 | .0330928 |
| NZL | di174 | -.483208 | .5601576 | -0.86 | 0.388 | -1.581097 | .6146806 |
| NER | di176 | -2.074278 | 1.089038 | -1.90 | 0.057 | -4.208753 | .060198 |
| NGA | di177 | -4.00408 | .6215601 | -6.44 | 0.000 | -5.222316 | -2.785845 |
| MKD | di179 | .2600903 | .6700448 | 0.39 | 0.698 | -1.053173 | 1.573354 |
| NOR | di181 | .3544461 | .5544624 | 0.64 | 0.523 | -.7322803 | 1.441173 |
| PAK | di187 | -4.854233 | 1.092393 | -4.44 | 0.000 | -6.995284 | -2.713182 |
| PAN | di189 | 1.618806 | .785423 | 2.06 | 0.039 | .0794048 | 3.158206 |
| PRY | di191 | 2.301772 | .7828837 | 2.94 | 0.003 | .7673479 | 3.836196 |
| PHL | di193 | -2.942737 | .5249556 | -5.61 | 0.000 | -3.971631 | -1.913842 |
| POL | di194 | -2.966485 | .5056445 | -5.87 | 0.000 | -3.95753 | -1.97544 |
| PRT | di195 | -2.365602 | .5239475 | -4.51 | 0.000 | -3.39252 | -1.338684 |
| PRI | di198 | -1.624167 | .7516663 | -2.16 | 0.031 | -3.097406 | -.1509286 |
| QAT | di199 | -1.174953 | .9968571 | -1.18 | 0.239 | -3.128758 | .7788507 |
| ROU | di200 | -1.679927 | .5076257 | -3.31 | 0.001 | -2.674855 | -.6849988 |
| RUS | di201 | -3.029887 | .5024997 | -6.03 | 0.000 | -4.014768 | -2.045005 |
| WSM | di203 | 2.451411 | .9549353 | 2.57 | 0.010 | .5797727 | 4.32305 |
| SMR | di204 | 2.92725 | 2.674149 | 1.09 | 0.274 | -2.313985 | 8.168485 |
| SAU | di206 | -3.926327 | .9946129 | -3.95 | 0.000 | -5.875732 | -1.976921 |
| SRB | di208 | -.0848547 | .5414342 | -0.16 | 0.875 | -1.146046 | .9763367 |
| SGP | di211 | .6638825 | 1.468818 | 0.45 | 0.651 | -2.214947 | 3.542712 |
| SVK | di213 | -1.133269 | .5029718 | -2.25 | 0.024 | -2.119076 | -.1474626 |
| SVN | di214 | -.4241972 | .5210687 | -0.81 | 0.416 | -1.445473 | .5970786 |
| ZAF | di218 | -3.238109 | .5257435 | -6.16 | 0.000 | -4.268547 | -2.20767 |
| ESP | di222 | -2.504755 | .5120025 | -4.89 | 0.000 | -3.508262 | -1.501249 |
| LKA | di223 | 5.157827 | 6.112417 | 0.84 | 0.399 | -6.82229 | 17.13794 |
| LCA | di225 | 5.493081 | .8499139 | 6.46 | 0.000 | 3.82728 | 7.158881 |
| SDN | di231 | -2.850605 | 1.056082 | -2.70 | 0.007 | -4.920488 | -.7807225 |
| SWE | di233 | -1.09979 | .5165584 | -2.13 | 0.033 | -2.112226 | -.087354 |
| CHE | di234 | -1.069547 | .5348447 | -2.00 | 0.046 | -2.117823 | -.0212706 |
| SYR | di235 | -1.912611 | .9072076 | -2.11 | 0.035 | -3.690705 | -.134517 |
| TJK | di236 | -1.829917 | .9563502 | -1.91 | 0.056 | -3.704329 | .0444951 |
| THA | di238 | 6.317738 | 7.673196 | 0.82 | 0.410 | -8.721449 | 21.35693 |
| TGO | di240 | .694561 | .799527 | 0.87 | 0.385 | -.872483 | 2.261605 |
| TTO | di242 | .4513594 | .830047 | 0.54 | 0.587 | -1.175503 | 2.078222 |
| TUN | di243 | -3.604198 | .9487296 | -3.80 | 0.000 | -5.463674 | -1.744722 |
| TUR | di244 | -4.423378 | .9270111 | -4.77 | 0.000 | -6.240286 | -2.60647 |
| TKM | di245 | -1.543608 | 1.089909 | -1.42 | 0.157 | -3.679791 | .5925751 |
| UGA | di248 | -2.214244 | .5443966 | -4.07 | 0.000 | -3.281241 | -1.147246 |
| UKR | di249 | -3.471952 | .5005916 | -6.94 | 0.000 | -4.453093 | -2.49081 |
| ARE | di250 | -2.285008 | 1.076207 | -2.12 | 0.034 | -4.394334 | -.1756822 |
| GBR | di251 | -2.86342 | .5036629 | -5.69 | 0.000 | -3.850581 | -1.876258 |
| USA | di252 | -2.935417 | .66021 | -4.45 | 0.000 | -4.229405 | -1.64143 |
| URY | di254 | .2005343 | .7832544 | 0.26 | 0.798 | -1.334616 | 1.735685 |
| UZB | di255 | -3.776549 | .9032068 | -4.18 | 0.000 | -5.546802 | -2.006296 |
| VEN | di257 | -3.203294 | .5330154 | -6.01 | 0.000 | -4.247985 | -2.158603 |
| VNM | di258 | 1.796163 | 4.339554 | 0.41 | 0.679 | -6.709207 | 10.30153 |
| ZMB | di263 | -.2460405 | .7729305 | -0.32 | 0.750 | -1.760956 | 1.268875 |
|  | _cons | -3.086898 | .6684895 | -4.62 | 0.000 | -4.397113 | -1.776682 |

**Table S4. SFA applied to GM and POLP with a COLLECTIVE perspective. No. observations = 601, No. groups = 100, non-significant μ (average inefficiency) = -0.365, non-significant η (increasing inefficiency) = −0.532, σu2 (similarity between countries) = 0.201, σv2 (similarity between countries over time) = 0.635. Bold = significant at 95%.**

| coucod | lgmpc | Coefficient | Std. err. | z | P>z | [95% conf. | interval] |
| --- | --- | --- | --- | --- | --- | --- | --- |
|  | lpolp | -1.178129 | .1681322 | -7.01 | 0.000 | -1.507663 | -.8485963 |
|  | gdps | 3.01e-07 | 5.44e-06 | 0.06 | 0.956 | -.0000104 | .000011 |
|  | ine | .1694792 | 1.081357 | 0.16 | 0.875 | -1.949941 | 2.2889 |
|  | budsoc | 5.514241 | 26.83927 | 0.21 | 0.837 | -47.08976 | 58.11824 |
|  | chrsoc | .2871811 | 1.841304 | 0.16 | 0.876 | -3.321708 | 3.89607 |
|  | hinsoc | 43.52711 | 111.7927 | 0.39 | 0.697 | -175.5825 | 262.6368 |
|  | islsoc | 1.617944 | 2.77828 | 0.58 | 0.560 | -3.827384 | 7.063272 |
|  | judsoc | 41.65735 | 41.45038 | 1.00 | 0.315 | -39.58389 | 122.8986 |
|  | geps | -1.392002 | .6498427 | -2.14 | 0.032 | -2.66567 | -.1183335 |
|  | gess | -.0710987 | .4011284 | -0.18 | 0.859 | -.8572959 | .7150986 |
|  | gets | -.4668596 | .295254 | -1.58 | 0.114 | -1.045547 | .1118277 |
|  | indsoc | .8977055 | .7907983 | 1.14 | 0.256 | -.6522307 | 2.447642 |
|  | dirsoc | -.0636127 | .3146447 | -0.20 | 0.840 | -.6803049 | .5530795 |
|  | consoc | 2.429391 | 3.55473 | 0.68 | 0.494 | -4.537753 | 9.396534 |
|  | teasoc | .0086731 | .7239033 | 0.01 | 0.990 | -1.410151 | 1.427497 |
| DZA | di3 | -3.103309 | 2.881208 | -1.08 | 0.281 | -8.750373 | 2.543755 |
| ARG | di9 | .2772972 | .7508117 | 0.37 | 0.712 | -1.194267 | 1.748861 |
| ARM | di10 | -.0300242 | .9269079 | -0.03 | 0.974 | -1.84673 | 1.786682 |
| AUS | di12 | 1.421743 | .8240097 | 1.73 | 0.084 | -.1932868 | 3.036772 |
| AUT | di13 | 1.17419 | .7114103 | 1.65 | 0.099 | -.220149 | 2.568528 |
| AZE | di14 | -1.75162 | 2.796624 | -0.63 | 0.531 | -7.232903 | 3.729662 |
| BHS | di15 | 2.850232 | 1.271702 | 2.24 | 0.025 | .3577419 | 5.342722 |
| BHR | di16 | -1.613005 | 7.622965 | -0.21 | 0.832 | -16.55374 | 13.32773 |
| BLR | di19 | .4111719 | .7377646 | 0.56 | 0.577 | -1.03482 | 1.857164 |
| BEL | di20 | .5751484 | .8319079 | 0.69 | 0.489 | -1.055361 | 2.205658 |
| BMU | di23 | 3.250681 | 1.451413 | 2.24 | 0.025 | .4059646 | 6.095398 |
| BWA | di27 | 1.14241 | 1.046998 | 1.09 | 0.275 | -.9096684 | 3.194489 |
| BRA | di28 | -1.182295 | .746827 | -1.58 | 0.113 | -2.646049 | .2814587 |
| BGR | di31 | -.0719934 | .7845483 | -0.09 | 0.927 | -1.60968 | 1.465693 |
| BDI | di33 | .0385088 | 1.08558 | 0.04 | 0.972 | -2.089188 | 2.166206 |
| CMR | di36 | -.5438977 | 1.003194 | -0.54 | 0.588 | -2.510123 | 1.422327 |
| CAN | di37 | .8296683 | .7281005 | 1.14 | 0.254 | -.5973825 | 2.256719 |
| CHL | di44 | -.2795733 | .8645074 | -0.32 | 0.746 | -1.973977 | 1.41483 |
| CHN | di45 | -3.57184 | 4.361542 | -0.82 | 0.413 | -12.1203 | 4.976625 |
| COL | di46 | -1.5817 | .8250918 | -1.92 | 0.055 | -3.19885 | .0354504 |
| CRI | di50 | .8224403 | 1.046348 | 0.79 | 0.432 | -1.228364 | 2.873245 |
| CIV | di51 | -1.844192 | 1.622697 | -1.14 | 0.256 | -5.024619 | 1.336235 |
| HRV | di52 | 1.737872 | .7172494 | 2.42 | 0.015 | .3320887 | 3.143655 |
| CUB | di53 | 1.030905 | .8571765 | 1.20 | 0.229 | -.6491297 | 2.71094 |
| CZE | di56 | .5472479 | 1.064771 | 0.51 | 0.607 | -1.539666 | 2.634161 |
| DNK | di57 | 2.45787 | .7830103 | 3.14 | 0.002 | .9231979 | 3.992542 |
| DMA | di59 | 5.028338 | 1.212551 | 4.15 | 0.000 | 2.651781 | 7.404895 |
| DOM | di60 | -.771339 | .7724981 | -1.00 | 0.318 | -2.285407 | .7427294 |
| ECU | di65 | -.3608875 | .8062729 | -0.45 | 0.654 | -1.941153 | 1.219378 |
| EGY | di66 | -4.641494 | 2.640949 | -1.76 | 0.079 | -9.817659 | .534671 |
| EST | di70 | 2.5894 | 1.098129 | 2.36 | 0.018 | .4371056 | 4.741694 |
| ETH | di72 | -2.31169 | 1.195751 | -1.93 | 0.053 | -4.655319 | .0319382 |
| FJI | di79 | -8.768133 | 31.04485 | -0.28 | 0.778 | -69.61492 | 52.07865 |
| FIN | di80 | 1.19624 | .7580406 | 1.58 | 0.115 | -.2894925 | 2.681972 |
| FRA | di82 | -.2362273 | .7391295 | -0.32 | 0.749 | -1.684894 | 1.21244 |
| GEO | di86 | .5834252 | .8577468 | 0.68 | 0.496 | -1.097728 | 2.264578 |
| DEU | di87 | .5027843 | .7133379 | 0.70 | 0.481 | -.8953323 | 1.900901 |
| GIB | di89 | -56.45931 | 62.51887 | -0.90 | 0.366 | -178.994 | 66.07542 |
| GRC | di90 | .5458767 | .7641533 | 0.71 | 0.475 | -.9518362 | 2.04359 |
| GRD | di92 | 2.810358 | 1.57779 | 1.78 | 0.075 | -.2820531 | 5.902769 |
| GTM | di94 | -.6941679 | 1.051964 | -0.66 | 0.509 | -2.75598 | 1.367645 |
| HKG | di102 | .238359 | 4.400248 | 0.05 | 0.957 | -8.385968 | 8.862686 |
| HUN | di103 | 1.835453 | .6998111 | 2.62 | 0.009 | .4638487 | 3.207058 |
| IND | di110 | -37.1352 | 81.6848 | -0.45 | 0.649 | -197.2345 | 122.9641 |
| IDN | di111 | -4.077125 | 2.351768 | -1.73 | 0.083 | -8.686506 | .5322564 |
| IRN | di112 | -3.264433 | 2.85192 | -1.14 | 0.252 | -8.854093 | 2.325227 |
| IRL | di114 | 1.084475 | .9096655 | 1.19 | 0.233 | -.6984362 | 2.867387 |
| ISR | di116 | -30.89076 | 30.07137 | -1.03 | 0.304 | -89.82957 | 28.04804 |
| ITA | di117 | -.3384067 | .6866785 | -0.49 | 0.622 | -1.684272 | 1.007458 |
| JAM | di118 | 2.779288 | .7110625 | 3.91 | 0.000 | 1.385631 | 4.172945 |
| JPN | di119 | -4.220531 | 15.15697 | -0.28 | 0.781 | -33.92765 | 25.48659 |
| JOR | di120 | -1.686383 | 2.912123 | -0.58 | 0.563 | -7.39404 | 4.021273 |
| KAZ | di121 | -1.635819 | 1.944516 | -0.84 | 0.400 | -5.447 | 2.175362 |
| KEN | di122 | -.9729357 | .6877307 | -1.41 | 0.157 | -2.320863 | .3749918 |
| PRK | di124 | -2.798176 | 1.879726 | -1.49 | 0.137 | -6.482372 | .8860192 |
| KOR | di125 | -1.093311 | 6.720546 | -0.16 | 0.871 | -14.26534 | 12.07872 |
| XKX | di126 | .6054741 | 2.742757 | 0.22 | 0.825 | -4.77023 | 5.981178 |
| LVA | di134 | 1.822425 | .7699214 | 2.37 | 0.018 | .3134069 | 3.331443 |
| LTU | di141 | 1.296202 | .7947162 | 1.63 | 0.103 | -.2614129 | 2.853817 |
| MEX | di156 | -1.935164 | .8200186 | -2.36 | 0.018 | -3.542371 | -.3279571 |
| MNG | di164 | -1.145653 | 14.61151 | -0.08 | 0.938 | -29.78369 | 27.49239 |
| MAR | di166 | -3.119277 | 2.894789 | -1.08 | 0.281 | -8.79296 | 2.554406 |
| MOZ | di167 | -1.902845 | 1.415151 | -1.34 | 0.179 | -4.67649 | .8708004 |
| NLD | di172 | 1.626668 | .7798417 | 2.09 | 0.037 | .0982062 | 3.155129 |
| NZL | di174 | 2.003839 | .8252036 | 2.43 | 0.015 | .3864693 | 3.621208 |
| NGA | di177 | -3.434524 | 1.854776 | -1.85 | 0.064 | -7.069818 | .200769 |
| NOR | di181 | 2.61935 | .8133614 | 3.22 | 0.001 | 1.025191 | 4.213509 |
| PAK | di187 | -4.900825 | 2.938599 | -1.67 | 0.095 | -10.66037 | .8587239 |
| PAN | di189 | 1.618045 | 1.032783 | 1.57 | 0.117 | -.4061736 | 3.642263 |
| PHL | di193 | -3.119857 | .8136903 | -3.83 | 0.000 | -4.71466 | -1.525053 |
| POL | di194 | -.3342842 | .7374946 | -0.45 | 0.650 | -1.779747 | 1.111179 |
| PRT | di195 | -.1172469 | .7888573 | -0.15 | 0.882 | -1.663379 | 1.428885 |
| PRI | di198 | -1.339501 | 1.223978 | -1.09 | 0.274 | -3.738453 | 1.059452 |
| QAT | di199 | .7512175 | 2.334168 | 0.32 | 0.748 | -3.823668 | 5.326103 |
| ROU | di200 | .2674496 | 1.665762 | 0.16 | 0.872 | -2.997385 | 3.532284 |
| RUS | di201 | -.193404 | .7264093 | -0.27 | 0.790 | -1.61714 | 1.230332 |
| SRB | di208 | 1.179215 | .7466901 | 1.58 | 0.114 | -.2842702 | 2.642701 |
| SGP | di211 | -1.921072 | 7.236267 | -0.27 | 0.791 | -16.1039 | 12.26175 |
| SVK | di213 | .6954264 | .693761 | 1.00 | 0.316 | -.6643201 | 2.055173 |
| SVN | di214 | 1.871144 | .7587489 | 2.47 | 0.014 | .3840236 | 3.358265 |
| ZAF | di218 | -1.405278 | .7384788 | -1.90 | 0.057 | -2.85267 | .042114 |
| ESP | di222 | -.3420886 | .7513551 | -0.46 | 0.649 | -1.814717 | 1.13054 |
| LCA | di225 | 4.243859 | 1.073557 | 3.95 | 0.000 | 2.139727 | 6.347992 |
| SWE | di233 | 1.26849 | .7971992 | 1.59 | 0.112 | -.2939915 | 2.830972 |
| CHE | di234 | .7568584 | .7469153 | 1.01 | 0.311 | -.7070687 | 2.220786 |
| SYR | di235 | -2.08363 | 2.687981 | -0.78 | 0.438 | -7.351977 | 3.184717 |
| TJK | di236 | -1.396876 | 2.936726 | -0.48 | 0.634 | -7.152754 | 4.359001 |
| THA | di238 | -6.550342 | 23.45214 | -0.28 | 0.780 | -52.51569 | 39.415 |
| TTO | di242 | -7.533101 | 27.23482 | -0.28 | 0.782 | -60.91237 | 45.84616 |
| TUN | di243 | -2.185412 | 2.889952 | -0.76 | 0.450 | -7.849613 | 3.478789 |
| TUR | di244 | -3.360293 | 2.843728 | -1.18 | 0.237 | -8.933898 | 2.213311 |
| UGA | di248 | -1.386012 | .8225512 | -1.69 | 0.092 | -2.998183 | .2261585 |
| UKR | di249 | -.636953 | .7077343 | -0.90 | 0.368 | -2.024087 | .7501808 |
| ARE | di250 | -2.060489 | 6.982883 | -0.30 | 0.768 | -15.74669 | 11.62571 |
| GBR | di251 | -.3656738 | .7216555 | -0.51 | 0.612 | -1.780093 | 1.048745 |
| USA | di252 | -.1713275 | .701853 | -0.24 | 0.807 | -1.546934 | 1.204279 |
| UZB | di255 | -2.205965 | 2.766282 | -0.80 | 0.425 | -7.627778 | 3.215847 |
| VEN | di257 | -1.750702 | .8817229 | -1.99 | 0.047 | -3.478848 | -.0225574 |
| VNM | di258 | -4.94801 | 13.30718 | -0.37 | 0.710 | -31.02961 | 21.13359 |
|  | _cons | -.3635843 | 1.807718 | -0.20 | 0.841 | -3.906647 | 3.179478 |

**Table S5. SFA applied to TM and POLC with a COLLECTIVE perspective. No. observations = 853, No. groups = 128, non-significant μ (average inefficiency) = -0.784, non-significant η (increasing inefficiency) = −1.039, σu2 (similarity between countries) = 0.209, σv2 (similarity between countries over time) = 0.381. Bold = significant at 95%.**

| coucod | ltmpc | Coefficient | Std. err. | z | P>z | [95% conf. | interval] |
| --- | --- | --- | --- | --- | --- | --- | --- |
|  | lpolc | 1.121747 | .0433735 | 25.86 | 0.000 | 1.036736 | 1.206757 |
|  | gdps | -4.45e-06 | 3.46e-06 | -1.29 | 0.199 | -.0000112 | 2.34e-06 |
|  | ine | -2.052042 | .7213446 | -2.84 | 0.004 | -3.465851 | -.6382321 |
|  | budsoc | -24.12195 | 14.37035 | -1.68 | 0.093 | -52.28732 | 4.043422 |
|  | chrsoc | 1.773287 | 1.181206 | 1.50 | 0.133 | -.5418338 | 4.088407 |
|  | hinsoc | -52.83422 | 40.2264 | -1.31 | 0.189 | -131.6765 | 26.00808 |
|  | islsoc | 3.155997 | 1.74859 | 1.80 | 0.071 | -.2711756 | 6.583171 |
|  | judsoc | -13.34115 | 20.05518 | -0.67 | 0.506 | -52.64857 | 25.96627 |
|  | geps | -.2555276 | .2774904 | -0.92 | 0.357 | -.7993989 | .2883436 |
|  | gess | -.1818726 | .2577111 | -0.71 | 0.480 | -.6869771 | .3232319 |
|  | gets | .1831045 | .1972872 | 0.93 | 0.353 | -.2035713 | .5697803 |
|  | indsoc | .5721587 | .4495818 | 1.27 | 0.203 | -.3090055 | 1.453323 |
|  | dirsoc | -.4139561 | .1779756 | -2.33 | 0.020 | -.7627818 | -.0651304 |
|  | consoc | 2.962267 | 1.974194 | 1.50 | 0.133 | -.9070816 | 6.831616 |
|  | teasoc | .8849273 | .395129 | 2.24 | 0.025 | .1104887 | 1.659366 |
| AFG | di1 | -2.588979 | 1.853415 | -1.40 | 0.162 | -6.221606 | 1.043648 |
| DZA | di3 | -4.837018 | 1.802056 | -2.68 | 0.007 | -8.368983 | -1.305052 |
| ARG | di9 | -2.194864 | .5371144 | -4.09 | 0.000 | -3.247589 | -1.142139 |
| ARM | di10 | -.166214 | .5587481 | -0.30 | 0.766 | -1.26134 | .9289121 |
| AUS | di12 | -1.629134 | .5785403 | -2.82 | 0.005 | -2.763052 | -.4952161 |
| AUT | di13 | -1.042385 | .5153945 | -2.02 | 0.043 | -2.05254 | -.0322306 |
| AZE | di14 | -2.407846 | 1.752553 | -1.37 | 0.169 | -5.842786 | 1.027095 |
| BHS | di15 | 2.264577 | .7577607 | 2.99 | 0.003 | .7793935 | 3.749761 |
| BHR | di16 | 2.952801 | 3.024135 | 0.98 | 0.329 | -2.974395 | 8.879997 |
| BRB | di18 | .8750073 | 1.018238 | 0.86 | 0.390 | -1.120703 | 2.870718 |
| BLR | di19 | -1.555134 | .5277804 | -2.95 | 0.003 | -2.589565 | -.5207039 |
| BEL | di20 | -1.193474 | .5661623 | -2.11 | 0.035 | -2.303132 | -.0838167 |
| BMU | di23 | 5.374657 | .9720528 | 5.53 | 0.000 | 3.469468 | 7.279845 |
| BWA | di27 | 2.146072 | .6018827 | 3.57 | 0.000 | .966404 | 3.325741 |
| BRA | di28 | -3.392084 | .546626 | -6.21 | 0.000 | -4.463451 | -2.320717 |
| BGR | di31 | -1.439195 | .5468938 | -2.63 | 0.008 | -2.511087 | -.3673028 |
| BFA | di32 | -1.591782 | 1.269495 | -1.25 | 0.210 | -4.079946 | .8963812 |
| BDI | di33 | -.6135214 | .6431358 | -0.95 | 0.340 | -1.874044 | .6470016 |
| CMR | di36 | -1.986033 | .6722719 | -2.95 | 0.003 | -3.303662 | -.6684047 |
| CAN | di37 | -1.596034 | .534108 | -2.99 | 0.003 | -2.642866 | -.5492011 |
| CHL | di44 | -1.35136 | .5686046 | -2.38 | 0.017 | -2.465804 | -.2369152 |
| CHN | di45 | -.3392299 | 2.366589 | -0.14 | 0.886 | -4.977659 | 4.299199 |
| COL | di46 | -3.280829 | .5519706 | -5.94 | 0.000 | -4.362671 | -2.198986 |
| CRI | di50 | .4340363 | .652792 | 0.66 | 0.506 | -.8454124 | 1.713485 |
| CIV | di51 | -2.557345 | .9995797 | -2.56 | 0.011 | -4.516486 | -.5982053 |
| HRV | di52 | -.3532404 | .5226227 | -0.68 | 0.499 | -1.377562 | .6710813 |
| CUB | di53 | -.297151 | .6047861 | -0.49 | 0.623 | -1.48251 | .888208 |
| CYP | di55 | .5677583 | .7374222 | 0.77 | 0.441 | -.8775626 | 2.013079 |
| CZE | di56 | -.7845572 | .7307075 | -1.07 | 0.283 | -2.216718 | .6476033 |
| DNK | di57 | .1739268 | .5540724 | 0.31 | 0.754 | -.9120352 | 1.259889 |
| DMA | di59 | 4.885837 | .8823423 | 5.54 | 0.000 | 3.156478 | 6.615196 |
| DOM | di60 | -1.392101 | .5439486 | -2.56 | 0.010 | -2.458221 | -.3259813 |
| ECU | di65 | -1.875171 | .5794104 | -3.24 | 0.001 | -3.010794 | -.7395471 |
| EGY | di66 | -6.297637 | 1.643562 | -3.83 | 0.000 | -9.518959 | -3.076315 |
| ERI | di69 | -.5984703 | 1.228948 | -0.49 | 0.626 | -3.007164 | 1.810224 |
| EST | di70 | 1.378052 | .7322624 | 1.88 | 0.060 | -.0571555 | 2.813261 |
| ETH | di72 | -3.289616 | .770307 | -4.27 | 0.000 | -4.79939 | -1.779842 |
| FJI | di79 | 15.49638 | 11.19597 | 1.38 | 0.166 | -6.44733 | 37.44008 |
| FIN | di80 | -.2752348 | .5344968 | -0.51 | 0.607 | -1.322829 | .7723597 |
| FRA | di82 | -2.632827 | .539296 | -4.88 | 0.000 | -3.689828 | -1.575826 |
| GAB | di84 | -.4269015 | .8086599 | -0.53 | 0.598 | -2.011846 | 1.158043 |
| GEO | di86 | -.3498138 | .5893943 | -0.59 | 0.553 | -1.505005 | .8053778 |
| DEU | di87 | -2.441905 | .5268095 | -4.64 | 0.000 | -3.474432 | -1.409377 |
| GHA | di88 | -2.4974 | .8385817 | -2.98 | 0.003 | -4.14099 | -.8538101 |
| GIB | di89 | -24.70745 | 35.4879 | -0.70 | 0.486 | -94.26246 | 44.84756 |
| GRC | di90 | -2.041549 | .554227 | -3.68 | 0.000 | -3.127814 | -.9552843 |
| GRD | di92 | 3.042641 | .7676238 | 3.96 | 0.000 | 1.538126 | 4.547156 |
| GTM | di94 | -1.572586 | .6573716 | -2.39 | 0.017 | -2.86101 | -.2841609 |
| HKG | di102 | 3.768249 | 2.375422 | 1.59 | 0.113 | -.8874931 | 8.423991 |
| HUN | di103 | -.9020798 | .5267542 | -1.71 | 0.087 | -1.934499 | .1303394 |
| ISL | di105 | 2.597559 | .7104937 | 3.66 | 0.000 | 1.205017 | 3.990101 |
| IND | di110 | 32.65677 | 29.47738 | 1.11 | 0.268 | -25.11783 | 90.43137 |
| IDN | di111 | -4.979664 | 1.486147 | -3.35 | 0.001 | -7.892458 | -2.06687 |
| IRN | di112 | -4.276133 | 1.791188 | -2.39 | 0.017 | -7.786796 | -.7654697 |
| IRL | di114 | -1.166537 | .598478 | -1.95 | 0.051 | -2.339533 | .0064579 |
| ISR | di116 | 9.106491 | 14.63113 | 0.62 | 0.534 | -19.57 | 37.78298 |
| ITA | di117 | -2.701327 | .5130238 | -5.27 | 0.000 | -3.706835 | -1.695819 |
| JAM | di118 | 1.602296 | .5303764 | 3.02 | 0.003 | .5627773 | 2.641815 |
| JPN | di119 | 11.24003 | 8.104543 | 1.39 | 0.165 | -4.644586 | 27.12464 |
| JOR | di120 | -2.138369 | 1.790345 | -1.19 | 0.232 | -5.64738 | 1.370642 |
| KAZ | di121 | -3.558855 | 1.238009 | -2.87 | 0.004 | -5.985307 | -1.132402 |
| KEN | di122 | -1.94123 | .5172091 | -3.75 | 0.000 | -2.954941 | -.927519 |
| PRK | di124 | -1.263198 | 1.148687 | -1.10 | 0.271 | -3.514584 | .988187 |
| KOR | di125 | 4.561516 | 3.594484 | 1.27 | 0.204 | -2.483542 | 11.60657 |
| XKX | di126 | -.4690635 | 1.729008 | -0.27 | 0.786 | -3.857857 | 2.91973 |
| KWT | di127 | -1.473185 | 1.564199 | -0.94 | 0.346 | -4.538959 | 1.592588 |
| KGZ | di128 | -1.713532 | 1.578231 | -1.09 | 0.278 | -4.806807 | 1.379744 |
| LVA | di134 | .0685787 | .5366246 | 0.13 | 0.898 | -.9831862 | 1.120344 |
| LIE | di140 | 2.720453 | 1.964961 | 1.38 | 0.166 | -1.1308 | 6.571706 |
| LTU | di141 | -.2400157 | .5302694 | -0.45 | 0.651 | -1.279325 | .7992933 |
| MYS | di149 | 1.85568 | 2.951781 | 0.63 | 0.530 | -3.929704 | 7.641064 |
| MUS | di155 | 25.47143 | 18.13782 | 1.40 | 0.160 | -10.07804 | 61.02091 |
| MEX | di156 | -4.10563 | .5459441 | -7.52 | 0.000 | -5.17566 | -3.035599 |
| MDA | di162 | -.550869 | .5713764 | -0.96 | 0.335 | -1.670746 | .5690082 |
| MNG | di164 | 14.00998 | 7.74114 | 1.81 | 0.070 | -1.162379 | 29.18233 |
| MNE | di165 | 2.194583 | .9157822 | 2.40 | 0.017 | .399683 | 3.989483 |
| MAR | di166 | -4.887909 | 1.805795 | -2.71 | 0.007 | -8.427202 | -1.348616 |
| MOZ | di167 | -.504827 | .9091733 | -0.56 | 0.579 | -2.286774 | 1.27712 |
| NAM | di169 | 1.634055 | .6555172 | 2.49 | 0.013 | .3492651 | 2.918845 |
| NLD | di172 | -.5728036 | .559889 | -1.02 | 0.306 | -1.670166 | .5245587 |
| NZL | di174 | -.2184477 | .589951 | -0.37 | 0.711 | -1.37473 | .937835 |
| NER | di176 | -2.471987 | 1.85267 | -1.33 | 0.182 | -6.103153 | 1.159179 |
| NGA | di177 | -4.483753 | .9510034 | -4.71 | 0.000 | -6.347685 | -2.61982 |
| MKD | di179 | .1192936 | .8386498 | 0.14 | 0.887 | -1.52443 | 1.763017 |
| NOR | di181 | .3994015 | .5689528 | 0.70 | 0.483 | -.7157254 | 1.514528 |
| PAK | di187 | -5.203423 | 1.86521 | -2.79 | 0.005 | -8.859168 | -1.547678 |
| PAN | di189 | 1.428035 | .7935283 | 1.80 | 0.072 | -.1272523 | 2.983322 |
| PRY | di191 | 2.085156 | .8027817 | 2.60 | 0.009 | .5117329 | 3.658579 |
| PHL | di193 | -3.249484 | .5567299 | -5.84 | 0.000 | -4.340654 | -2.158313 |
| POL | di194 | -3.292038 | .5406149 | -6.09 | 0.000 | -4.351624 | -2.232453 |
| PRT | di195 | -2.465833 | .5411716 | -4.56 | 0.000 | -3.52651 | -1.405156 |
| PRI | di198 | -2.098052 | .7253304 | -2.89 | 0.004 | -3.519673 | -.6764302 |
| QAT | di199 | -.2454668 | 1.451266 | -0.17 | 0.866 | -3.089897 | 2.598963 |
| ROU | di200 | -.1753442 | 1.096361 | -0.16 | 0.873 | -2.324172 | 1.973483 |
| RUS | di201 | -3.321123 | .5399019 | -6.15 | 0.000 | -4.379311 | -2.262935 |
| WSM | di203 | 2.091153 | .928851 | 2.25 | 0.024 | .270638 | 3.911667 |
| SMR | di204 | 3.617556 | 2.645389 | 1.37 | 0.171 | -1.567312 | 8.802424 |
| SAU | di206 | -3.479713 | 1.691766 | -2.06 | 0.040 | -6.795512 | -.163913 |
| SRB | di208 | -.5340171 | .5427875 | -0.98 | 0.325 | -1.597861 | .5298269 |
| SGP | di211 | 6.605356 | 3.113875 | 2.12 | 0.034 | .5022738 | 12.70844 |
| SVK | di213 | -1.151279 | .5131261 | -2.24 | 0.025 | -2.156988 | -.1455708 |
| SVN | di214 | -.4391892 | .5313058 | -0.83 | 0.408 | -1.480529 | .6021511 |
| ZAF | di218 | -3.191507 | .5430933 | -5.88 | 0.000 | -4.25595 | -2.127064 |
| ESP | di222 | -2.583756 | .5423474 | -4.76 | 0.000 | -3.646737 | -1.520775 |
| LKA | di223 | 22.51323 | 11.23048 | 2.00 | 0.045 | .5018941 | 44.52457 |
| LCA | di225 | 5.29849 | .8141709 | 6.51 | 0.000 | 3.702744 | 6.894235 |
| SDN | di231 | -3.286178 | 1.767814 | -1.86 | 0.063 | -6.75103 | .1786738 |
| SWE | di233 | -.515983 | .559119 | -0.92 | 0.356 | -1.611836 | .5798701 |
| CHE | di234 | -.8892795 | .5372783 | -1.66 | 0.098 | -1.942325 | .1637666 |
| SYR | di235 | -2.34424 | 1.666281 | -1.41 | 0.159 | -5.610091 | .9216118 |
| TJK | di236 | -2.136966 | 1.795368 | -1.19 | 0.234 | -5.655822 | 1.38189 |
| THA | di238 | 19.21264 | 12.55027 | 1.53 | 0.126 | -5.385443 | 43.81072 |
| TGO | di240 | 1.113774 | .8974095 | 1.24 | 0.215 | -.6451164 | 2.872664 |
| TTO | di242 | 13.96423 | 9.786548 | 1.43 | 0.154 | -5.217049 | 33.14552 |
| TUN | di243 | -3.753675 | 1.81198 | -2.07 | 0.038 | -7.30509 | -.2022607 |
| TUR | di244 | -4.940335 | 1.782385 | -2.77 | 0.006 | -8.433745 | -1.446925 |
| TKM | di245 | -1.55424 | 1.849286 | -0.84 | 0.401 | -5.178774 | 2.070294 |
| UGA | di248 | -2.529258 | .5862781 | -4.31 | 0.000 | -3.678342 | -1.380174 |
| UKR | di249 | -3.531914 | .5169845 | -6.83 | 0.000 | -4.545185 | -2.518643 |
| ARE | di250 | 2.11392 | 2.868541 | 0.74 | 0.461 | -3.508317 | 7.736157 |
| GBR | di251 | -2.674559 | .5206108 | -5.14 | 0.000 | -3.694938 | -1.654181 |
| USA | di252 | -3.404017 | .5265439 | -6.46 | 0.000 | -4.436024 | -2.372009 |
| URY | di254 | .4914211 | .8089552 | 0.61 | 0.544 | -1.094102 | 2.076944 |
| UZB | di255 | -4.000291 | 1.729856 | -2.31 | 0.021 | -7.390746 | -.609835 |
| VEN | di257 | -3.51559 | .5609993 | -6.27 | 0.000 | -4.615129 | -2.416052 |
| VNM | di258 | 9.700173 | 7.071989 | 1.37 | 0.170 | -4.160671 | 23.56102 |
| ZMB | di263 | -.2793411 | .7838317 | -0.36 | 0.722 | -1.815623 | 1.256941 |
|  | _cons | -4.434657 | 1.134111 | -3.91 | 0.000 | -6.657474 | -2.21184 |
